# The mechanism of breast cancer stem cells and tumor microenvironment promoting radioresistance in breast cancer and its intervention strategies

**DOI:** 10.3389/fimmu.2026.1786163

**Published:** 2026-09-09

**Authors:** Ying Zhao, Xiaohan Liu, Xin Bai, Jianming Tang

**Affiliations:** 1 The First School of Clinical Medicine, Lanzhou University, Lanzhou, Gansu, China; 2 Department of Radiation Oncology, The First Hospital of Lanzhou University, Lanzhou University, Lanzhou, Gansu, China

**Keywords:** breast cancer, breast cancer stem cells, combination radiotherapy, immunotherapy, radioresistance, targeted therapy, tumor microenvironment

## Abstract

**Background:**

Breast cancer (BC) is the most common malignant tumor among women worldwide. Radiotherapy (RT) is a primary treatment modality; however, local recurrence driven by radioresistance frequently undermines its efficacy.

**Mechanisms:**

Growing evidence indicates that breast cancer stem cells (BCSCs) and the tumor microenvironment (TME) cooperatively regulate multiple signaling pathways, thereby reducing RT efficacy. BCSCs exhibit intrinsic radioresistance through enhanced DNA repair capacity (Section 2.2.1), maintenance of redox homeostasis via the NRF2-KEAP1 and HIF-1α axes (Section 2.2.2), dysregulated cell cycle checkpoints (Section 2.2.3), and epithelial-mesenchymal transition (EMT) (Section 2.2.4). Concurrently, TME components—particularly cancer-associated fibroblasts (CAFs), tumor-associated macrophages (TAMs), regulatory T cells (Tregs), and myeloid-derived suppressor cells (MDSCs)—construct a protective niche that sustains BCSCs, promotes immune evasion, and triggers post-RT recurrence.

**Subtype-specific heterogeneity:**

The molecular subtyping of BC (Luminal A/B, HER2+, and triple-negative breast cancer (TNBC)) profoundly influences radiosensitivity and resistance mechanisms, necessitating subtype-specific therapeutic strategies (Section 2.1.2 and 3.1.1).

**Intervention strategies and challenges:**

To overcome radioresistance, we discuss emerging combination approaches, including RT with immune checkpoint inhibitors (ICIs), BCSCs-directed agents, metabolic interventions, and nanodelivery systems. We critically appraise translational barriers, including the paucity of validated biomarkers, the plasticity of BCSCs, and the toxicity profiles of multimodal regimens.

**Conclusion:**

By explicitly distinguishing preclinical from clinical evidence and identifying knowledge gaps between mechanistic insights and clinical application, this review provides a framework for rational trial design and precision RT in BC.

## 1 Introduction

BC is one of the most common cancers among women. RT is a standard treatment for it, but the existence of radioresistance often leads to poor treatment outcomes and local recurrence in some patients. Studies have shown that RT may enhance the migration and invasion abilities of BC cells (1). Recurrence after RT may result from the inherent resistance of tumor cells or the development of radioresistance in particular cell subsets (2).

Cancer stem cells (CSCs) are a subset of cells present within tumors, possessing the abilities of self-renewal, differentiation, and DNA repair. Due to these characteristics, tumors become more invasive, accelerating cancer progression and becoming the main factor contributing to the development of resistance to conventional therapies, including RT (3).

In the TME, cells such as mesenchymal stem cells (MSCs), CAFs, extracellular matrix (ECM) components, immune cells, and vascular systems can secrete diverse factors. These signaling molecules interact and influence each other, enhancing the proliferative capacity of BCSCs, recruiting different immune cells into the TME, and driving their specific “reprogramming” that alters the local immune response, thereby promoting the formation of radioresistance.

To overcome the radioresistance of BC, it is urgent to identify new targets and improve treatment outcomes. Targeting the immune system has become increasingly of interest as a promising treatment approach, especially through combined therapies that regulate immune responses and improve RT efficiency. Currently, several methods are being studied, including ICIs, cytokine therapy, and nanomedicines targeting the TME. Additionally, drugs that reduce the recruitment of immunosuppressive cells, such as MDSCs, Tregs, and TAMs, are also expected to enhance the efficacy of RT. The BCSCs-TME axis has become a major area of study for clarifying the processes underlying RT failure in BC.

To enhance the clinical translational relevance of this review, we clearly divided the supporting evidence into two levels: the preclinical evidence obtained from *in vitro* cell line experiments and *in vivo* animal models, as well as the clinical evidence derived from human trials (including I-III phase studies, real-world cohorts, and FDA-approved therapies). This distinction is of crucial importance, as although many mechanisms related to specific pathways have been identified, no clinical targets have been found that can overcome the radioresistance of breast cancer patients yet.

The molecular subtyping of BC (Luminal A, Luminal B, HER2+, and TNBC) not only determines tumor biology and treatment strategies but also profoundly influences radiosensitivity and mechanisms of resistance. Studies have demonstrated significant differences in post-RT prognosis across subtypes: Luminal A exhibits the most favorable overall prognosis, whereas HER2+ and TNBC subtypes display higher intrinsic radioresistance and a higher risk of local recurrence (4). These differences are partially attributed to variations in BCSC proportions, TME immune composition, and activation status of key signaling pathways among subtypes. For instance, TNBC and HER2+ subtypes exhibit significantly higher CD8^+^ T-cell infiltration than hormone receptor-positive subtypes; however, TNBC also harbors elevated levels of MDSCs and M2-TAMs, thereby establishing a unique immunosuppressive microenvironment (5, 6). Therefore, understanding subtype-specific BCSCs-TME interaction networks is essential for developing precision RT combination strategies.

This review discusses the complex mechanisms of radioresistance in BC, focusing on the characteristics of BCSCs and the recent progress in TME metabolism on radioresistance. It also discusses new combined strategies to overcome radioresistance and the challenges of applying these methods in clinical practice. The hope is to provide new ideas and insights to overcome BC radioresistance by developing novel targeted treatments.

## 2 Molecular mechanisms of BCSCs and radioresistance

### 2.1 Characterization and markers of BCSCs

Extensive evidence indicates that BCSCs are closely associated with the initiation, metastasis, invasion, and therapeutic resistance of BC (7–11). BCSCs are considered a pivotal element in tumor heterogeneity due to their ability to self-renew and differentiate into diverse cell types that constitute the tumor, thereby promoting tumor progression and metastasis (12–14).

BCSCs share biological characteristics with normal stem cells and play a central role in driving tumorigenesis, progression, metastasis, and treatment resistance. Their capacity for self-renewal can preserve tumor heterogeneity. They also express important transcription factors such as OCT4, NANOG, and SOX2. These factors not only regulate the self-renewal process but also promote tumor resistance to RT and chemotherapy (15, 16).

CD44, CD24, and ALDH1 are commonly used for BCSCs identification. The CD44^+^/CD24^−^ phenotype often indicates enhanced tumorigenic potential and poorer clinical outcome (17–20). Elevation of ALDH1 activity has also been identified as a functional marker of cells with a high propensity for self-renewal and tumor initiation (21, 22). Other markers, including ABCG2, CD133, CD49f, LGR5, SSEA-3, CD70, and PROCR have also been reported; however, their specificity and consistency across studies are still restricted (23–28).

#### 2.1.1 Subtype-specific distribution of BCSCs markers

The distribution of BCSCs markers across molecular subtypes exhibits significant heterogeneity, directly impacting stemness properties and therapeutic resistance in each subtype. A study analyzed 466 invasive BC cases and found that the CD44^+^/CD24^-^/low phenotype was most enriched in basal-like tumors (P = 0.039 vs. Luminal A; P = 0.008 vs. Luminal B), whereas ALDH1^+^ cells were more prevalent in HER2+ and basal-like subtypes. Notably, CD44^+^/CD24^-^/low and ALDH1^+^ may identify BCSCs at different differentiation stages: the former is more common in basal-like carcinomas (likely originating from primitive mammary stem cells), while the latter marks basal-like and HER2+ tumors, potentially arising from luminal-committed progenitor cells (7).

At the cell line level, luminal-type (MCF-7, T47D) and HER2+ (SkBr3, BT474) lines are predominantly composed of CD44^-^/CD24^+^ epithelial cells, whereas basal/mesenchymal-type (MDA-MB-231, BT-549) are enriched for CD44^+^/CD24^-^/low BCSCs (7, 29). ALDH1 activity is highest in HER2+ and basal-like epithelial cell lines and lowest in luminal-type lines. This subtype-specific marker distribution suggests that BCSC-targeting strategies must account for unique surface marker profiles in different subtypes.

Clinically, the CD44^+^/CD24^-^/low phenotype correlates with worse disease-free survival (DFS) and overall survival (OS) in basal-like tumors, particularly in ER- patients, where this phenotype is associated with significantly shortened post-metastasis OS (30). Thus, subtype-specific BCSCs marker distribution not only reflects tumor heterogeneity but also serves as an important predictor of RT response and recurrence risk.

Controversies and evidence gaps in BCSCs marker prognostication. Despite the biological plausibility of BCSCs markers, their prognostic utility remains contentious and is characterized by significant inter-study heterogeneity. Large-cohort analyses of invasive ductal carcinoma have demonstrated that the CD44^+^/CD24^-^ phenotype fails to maintain independent prognostic significance in multivariate models and lacks a robust association with OS (8). These findings stand in stark contrast to studies endorsing CD44^+^/CD24^-^ as a reliable predictor of poor outcomes (Idowu et al., 2012; Yang et al., 2016), underscoring the critical lack of methodological standardization across laboratories. Current definitions of CD44 positivity vary arbitrarily from ≥5% to ≥10% of immunostained cells, while CD24 negativity is inconsistently defined as either “complete absence” or “≤25% positivity,” severely compromising cross-study comparability (8). Compounding this complexity, evidence suggests a subtype-dependent functional dichotomy: the CD44^+^/CD24^-^ phenotype correlates with prolonged disease-free survival (DFS) in ER+ disease but portends shorter post-metastatic OS in ER- tumors (29), indicating a context-dependent rather than universal role. Furthermore, quantitative assessments reveal that the CD44/CD24 expression ratio—a more accurate proxy for stemness than binary classification—exhibits poor concordance with ALDH1 activity, with overlapping populations accounting for less than 1% of cells. These discrepancies underscore a critical evidence gap: current BCSCs markers may capture distinct, non-overlapping subpopulations at different differentiation stages, and their prognostic utility is confounded by molecular subtype, treatment context, and analytical standardization. Future studies must establish harmonized scoring systems and validate markers within subtype-stratified cohorts to resolve these contradictions. The controversy regarding ALDH1 as a functional marker for BCSCs is also significant: although ALDH1^+^ cells exhibit stronger spheroid formation ability and tumorigenicity *in vitro*, their expression varies greatly among different molecular subtypes (high expression in HER2+ and basal-like subtypes, and almost absent in Luminal type), which limits its application as a cross-subtype universal marker. Additionally, standardization of ALDH1 activity detection methods is low, and differences in flow cytometry settings across laboratories make it difficult to compare results (22, 30).

### 2.2 Mechanisms of BCSCs mediating radioresistance

#### 2.2.1 DNA repair pathway

Ionizing radiation (IR) induces DNA double-strand breaks (DSBs), thereby activating the DNA damage response (DDR) pathway. According to studies, BCSCs exhibit much higher expression of DNA repair-related genes than non-stem cells, which is one of the fundamental processes underlying their increased radioresistance (31, 32). Also, activating pro-survival signaling pathways such as Wnt/β-catenin, Notch, Hedgehog, TGF-β, and PI3K/AKT/mTOR makes BCSCs more resistant to RT (33, 34).

Preclinical evidence has firmly established that BCSCs exhibit enhanced homologous recombination repair (HRR) and non-homologous end joining (NHEJ) capabilities, with greater reliance on the G2/M checkpoint due to p53/p21 dysfunction (31, 32, 35–38). Clinically, however, direct targeting of checkpoint kinases (CHK1/2, WEE1, PLK1) remains investigational. The most advanced clinical application of DDR targeting in BC is PARP inhibition (olaparib, niraparib, talazoparib), which has demonstrated synthetic lethality in BRCA-mutant settings. Their specific efficacy in eradicating radioresistant BCSCs is currently being evaluated in early-phase trials (RADIOPARP, NCT03109080; TOPACIO/KEYNOTE-162, NCT02657889) (39–41).

Different molecular subtypes exhibit distinct dependencies on DNA repair pathways. TNBC and basal-like BC frequently harbor BRCA1/2 mutations or functional deficiencies, resulting in impaired HRR capacity and consequent sensitivity to PARP inhibitors (e.g., olaparib) (4). The OlympiA trial confirmed that olaparib adjuvant therapy significantly improves invasive disease-free survival (IDFS) in gBRCA-mutated high-risk early BC patients (42). In contrast, Luminal-type BC relies more on NHEJ and base excision repair (BER), with relatively preserved p53/p21 signaling axis and more intact G1/S checkpoint function. HER2+ BC displays unique radioresistance mechanisms, including NF-κB-mediated transactivation of the HER2 promoter, β-catenin upregulation during EMT, and FAK-mediated signaling activation. These subtype-specific DNA repair characteristics determine combination therapy selection: PARP inhibitor plus RT in TNBC demonstrates 65% 3-year event-free survival (EFS), whereas different sensitization targets must be explored in Luminal-type disease.

Translational gap in targeting DNA repair for radiosensitization. While preclinical evidence has firmly established that BCSCs exhibit enhanced HRR and NHEJ capabilities, with greater reliance on the G2/M checkpoint due to p53/p21 dysfunction (35–38), the clinical translation of these findings remains limited. Direct targeting of checkpoint kinases (CHK1/2, WEE1, PLK1) in BC is largely investigational, with no Phase III trials demonstrating survival benefit. The most advanced clinical application of DDR targeting in BC comes from PARP inhibitors (olaparib, niraparib, talazoparib), which have demonstrated synthetic lethality in BRCA-mutated settings (42) (39). However, their specific efficacy in eradicating radioresistant BCSCs—rather than bulk tumor cells—has not been prospectively validated in biomarker-driven trials. A critical unresolved question is whether PARP inhibitor-mediated radiosensitization selectively targets BCSCs or merely exploits general HRR deficiency in the entire tumor cell population. Furthermore, the optimal sequencing of PARP inhibitors relative to RT (concurrent vs. sequential) and the impact of fractionation on BCSC-specific DNA repair dynamics remain undefined. The RADIOPARP trial (NCT03109080) and TOPACIO/KEYNOTE-162 (NCT02657889) are exploring these questions, but results are pending (43). The lack of validated biomarkers to identify patients whose BCSCs are particularly dependent on HRR rather than NHEJ represents a major barrier to the precision application of DDR-targeted radiosensitization.

#### 2.2.2 Enhanced antioxidant stress capabilities

The existing evidence indicates that the lower reactive oxygen species (ROS) levels in BCSCs, together with the antioxidant defense system, constitute the key mechanism for their adaptive radioresistance (44); therefore, simultaneously targeting the NRF2 and HIF-1α signaling axes represents a potential therapeutic strategy. Elucidating the molecular basis of enhanced antioxidant capacity in BCSCs is critical for overcoming radioresistance and improving the clinical efficacy of BC. In terms of antioxidant defense, BCSCs can efficiently neutralize RT-induced ROS, thereby attenuating DNA damage and apoptotic signals. This process is primarily mediated by the continuous activation of the NRF2-KEAP1 antioxidant pathway (45). Under normal circumstances, KEAP1 can bind NRF2 and promote its ubiquitination-mediated degradation; however, in BCSCs, this regulatory mechanism is disrupted, resulting in the stabilization of NRF2 and its translocation to the nucleus. Activated NRF2 subsequently forms a heterodimer with the small Maf protein and binds to the antioxidant response elements (AREs) in the target genes, thereby upregulating the expression of downstream genes such as heme oxygenase-1 (HO-1), NAD(P)H: quinone oxidoreductase 1 (NQO1), and glutamate-cysteine ligase catalytic subunit (GCLC). These proteins collectively enhance intracellular glutathione (GSH) levels and antioxidant stress responses (46, 47). It is particularly noteworthy that recent studies have shown that the epigenetic regulatory factor ZMYND8 can act as a co-activator of NRF2, assisting it in binding to the AREs of antioxidant genes and significantly promoting the transcription of genes such as GSTP1, GPX2, and GCLM in BCSCs (48).

Meanwhile, the expression and activity of key antioxidant enzymes in BCSCs, such as superoxide dismutase 2 (SOD2) and catalase (CAT), are relatively high, thereby efficiently eliminating superoxide anions and hydrogen peroxide (49). Additionally, BCSCs further enhance their antioxidant state through metabolic reprogramming: using oxidative phosphorylation and fatty acid oxidation to generate NADPH, which supports GSH regeneration and the thioredoxin system; and by enhancing mitochondrial autophagy to selectively clear dysfunctional mitochondria, thereby reducing the generation of endogenous ROS (50). Concurrently, the hypoxic state is a characteristic of the BC TME and can activate hypoxia-inducible factor-1α (HIF-1α) and enhance the antioxidant capacity of BCSCs (51). Although hypoxia can limit oxygen supply, paradoxically, chronic hypoxia increases mitochondrial ROS production through complex III dysfunction, thereby prolonging the half-life of superoxide disulfide radicals and increasing electron leakage to oxygen. To counteract the oxidative stress induced by hypoxia, HIF-1α can upregulate the expression of antioxidant enzymes such as glutathione S-transferase (GSTs), superoxide dismutase (SOD), and catalase, thereby enhancing the cell’s ability to clear RT-induced ROS (52, 53).

What is particularly crucial is that HIF-1α and NRF2 form a positive feedback regulatory loop, jointly constituting the antioxidant defense system of BCSCs. On the one hand, NRF2 can directly bind to the AREs in the promoter region of the HIF1A gene, promoting the expression of HIF-1α; on the other hand, the target genes of HIF-1α, such as NQO1 and thioredoxin-1 (TRX1), can stabilize HIF-1α protein by inhibiting the activity of prolyl hydroxylase (PHD) (54). This HIF-NRF2 interaction enables BCSCs to maintain lower ROS levels than non-stem cells. Moreover, HIF-1α promotes a metabolic shift toward the glycolytic pathway, reducing mitochondrial oxygen consumption and ROS production, and upregulating the serine synthesis pathway mediated by phosphoglycerate dehydrogenase (PHGDH), thereby providing raw materials for the generation of GSH and NADPH (55).

Additionally, the cystine/glutamate transporter xCT (SLC7A11) is often overexpressed in BCSCs, promoting cystine uptake to drive GSH synthesis and conferring resistance to ferroptosis (a ROS-dependent regulatory cell death) (56). Recent studies have shown that radiation can induce SLC7A11 expression, and its overexpression can promote radiation resistance, whereas inhibiting SLC7A11 can enhance the radiation sensitivity of BCSCs (48, 57). In conclusion, these interrelated mechanisms - including the continuous activation of NRF2, hypoxia-induced HIF-1α signaling transduction, the collaborative positive feedback regulation of the two, metabolic adaptation, and ferroptosis resistance - jointly establish an oxidative-reductive environment favorable for BCSCs, enabling them to resist radiation-induced oxidative stress, survive in the treatment of damage, and drive tumor recurrence. Therefore, simultaneously targeting the NRF2 and HIF-1α signaling axes represents a potential therapeutic strategy that may enhance the radiosensitivity of BCSCs and prevent treatment failure (54).

Despite the compelling preclinical rationale for dual targeting of HIF-1α and NRF2. First, HIF-1α inhibitors such as digoxin, acriflavine, and EZN-2968 have shown limited efficacy in phase I/II trials for advanced cancers, with significant cardiovascular toxicity profiles (54). Second, the degree of NRF2 pathway activation varies markedly across molecular subtypes: TNBC frequently exhibits KEAP1 functional loss or constitutive NRF2 activation, whereas Luminal-type BC shows relatively lower basal NRF2 activity. HER2+ BC may stabilize NRF2 indirectly through HER2-PI3K-Akt-mTOR signaling. This subtype-specific heterogeneity implies that NRF2-targeted radiosensitization would require companion biomarkers and individualized dosing strategies. Third, no clinical-grade NRF2 inhibitor with adequate specificity for BCSCs over normal tissue stem cells has been developed to date. The potent antioxidant function of NRF2 in hematopoietic stem cells (HSCs) and intestinal crypt stem cells raises significant toxicity concerns: indiscriminate NRF2 inhibition could impair normal stem cell maintenance, leading to myelosuppression and mucositis (58). Fourth, the positive feedback between HIF-1α and NRF2 implies that dual targeting may be necessary for efficacy, yet no clinical trial has tested this combination in BC. The development of hypoxia-activated prodrugs (e.g., evofosfamide, TH-302) represents an alternative strategy, but these agents have shown limited efficacy in Phase II trials for other solid tumors, and their activity against BCSCs specifically has not been evaluated.

#### 2.2.3 Cell cycle regulation

IR has been demonstrated to induce DNA damage response (DDR) by activating DNA damage checkpoints. This halts the cell cycle at specific intervals (such as G1/S, S phase, early G2, and late G2) to facilitate DNA repair. However, in tumor cells, the function of DNA damage checkpoints may be impaired by frequent mutations in key checkpoint-associated genes, leading to reduced fidelity of DNA repair and increased genomic instability.

In BC, RT can synergistically enhance BCSC characteristics through multiple mechanisms. Firstly, radiation can induce the transformation of non-differentiated cancer cells into BCSCs through processes such as EMT. Studies have shown that the ALDH1^-^ non-stem cell population treated with ionizing radiation can exhibit dose-dependent enrichment of ALDH1^+^ cells (59). These cells have higher expression levels of stem cell-related genes (Sox2, Oct4, Nanog), better sphere-forming ability, and stronger tumor-forming ability *in vivo*. This phenotypic transformation is closely related to the activation of pathways such as Notch and NF-κB, which, by upregulating EMT transcription factors such as Snail, confer stem-cell characteristics on non-stem cells. BCSCs exhibit more robust activation of DNA damage checkpoints and stronger DNA repair activity than non-stem cells, leading to their radioresistance (35).

In addition, cells at different stages of the cell cycle have varying sensitivities to RT: cells in mitosis or the G2 phase are usually more sensitive, whereas cells in late S phase appear radioresistant. This difference in sensitivity is closely related to the dynamics of DNA damage repair capacity during the cell cycle, which in turn affects the cellular response to subsequent RT.

Radiation mainly activates ATM/ATR kinases by inducing double-strand DNA breaks. Activated ATM/ATR phosphorylates and activates downstream effectors CHK1/CHK2, thereby inhibiting the activity of the CDC25 phosphatase family (especially CDC25C). This keeps the CDK1/Cyclin B1 complex in an inhibitory phosphorylated state, effectively blocking the cell cycle at the G2/M checkpoint and preventing damaged cells from entering mitosis.

In BCSCs, dysfunction or weakening of the p53/p21 signaling axis often renders G1/S checkpoint function incomplete. This makes BCSCs more dependent on the G2/M checkpoint for DNA repair after RT damage. It is notable that although G2/M phase cells are sensitive to RT, after radiation “screening”, a group of BCSCs with stronger repair ability and greater radiation resistance survives. These cells can fine-tune key cell cycle regulators such as Wee1 kinase, CDC25 family members, and PLK1, ultimately overcoming the G2/M checkpoint block and re-entering the cell cycle to complete repair and continue proliferation.

The surviving BCSCs generally exhibit stronger radioresistance, which is particularly significant in the residual cells after RT. These cells tend to converge towards the S phase in the subsequent cycle, ultimately leading to a relative enrichment of the residual cell population in the S phase after RT. S-phase cells have unique advantages for efficient HRR: the single-stranded DNA region at the replication fork is covered by replication protein A, providing a key platform for the recruitment and complete activation of the ATRIP-ATR complex, thereby initiating the high-fidelity HRR mediated by RAD51 chain invasion. Additionally, the high level of Cyclin A-CDK2 kinase activity in S-phase cells not only drives DNA replication but also promotes the proper assembly and functional execution of the repair complex by phosphorylating BRCA1, PALB2, and other core HRR proteins. This unique molecular environment enables surviving BCSCs to repair radiation-induced DSBs with high fidelity, thereby more accurately repairing these breaks and maintaining the genomic stability and self-renewal ability of BCSCs (36–38).

Abnormalities in the cell cycle regulatory network further exacerbate this process. Studies have shown that aberrant expression of cell cycle-related proteins (e.g., Cyclin D1, CDK4/6) may promote the survival and proliferation of BCSCs after RT. In BC, RT may reactivate dormant BCSCs, causing them to re-enter the cell cycle. This can make tumors grow back faster and be harder to treat (60–62).

The cell-cycle dynamics described above—particularly S-phase enrichment and G2/M checkpoint dependency—are derived primarily from preclinical radiation fractionation studies (36–38). Clinical evidence linking specific cell-cycle regulator expression (e.g., Cyclin D1, CDK4/6) to post-RT BCSCs enrichment remains largely correlative. At present, there are insufficient prospective clinical trials specifically designed to target cell-cycle regulatory factors to overcome radioresistance driven by BCSCs. Moreover, the evidence regarding the relationship between the expression of specific cell-cycle regulatory factors and the enrichment of BCSCs after RT in human BC samples is largely correlational rather than causal. Although *in vitro* models have confirmed that inhibiting Wee1, CDC25, and PLK1 can enhance the sensitivity of BCSCs to RT by blocking the G2/M checkpoint, these findings largely derive from cell lines and patient-derived xenograft (PDX) studies. The key point is that PDX models lack a functional human immune system and cannot simulate cytokine signaling (such as IL-6 and TGF-β) mediated by the TME in the body, which regulates the dynamics of cell cycle checkpoints. Especially in the context of the frequently used fractionated RT regimens in clinical practice, whether drug-induced G2/M checkpoint blockade can specifically eliminate BCSCs without causing disproportionate toxic effects on normal tissue stem cells remains an issue that needs to be addressed and verified in models with intact immune function and subtype specificity.

#### 2.2.4 EMT

EMT is characterized by the downregulation of epithelial markers (such as E-cadherin and Claudin) and the gain of mesenchymal markers (such as vimentin and N-cadherin) (63), and activation of EMT enhances tumor cell invasion and metastasis (64, 65). In BC and other cancers, radiation induces EMT, which increases cell invasion metastasis. Evidence indicates that RT may enhance the metastatic potential of primary tumors (66–69). ROS promote RT-induced EMT by activating key transcription factors (such as Snail, HIF-1, ZEB1, and STAT3) within critical signaling pathways, including TGF-β, Wnt, Hedgehog, Notch, G-CSF, EGFR/PI3K/Akt, and MAPK.

Studies have found that α-actinin-4 (ACTN4) can induce EMT by activating the Akt pathway, thereby promoting BC progression and enhancing radioresistance. Inhibiting ACTN4 can reverse this phenotype (70). BC cells with radioresistance often exhibit chemotherapy resistance as well, and enhanced migratory ability is closely related to EMT and upregulation of related factors (71). It is noteworthy that RT itself can induce EMT, making targeting this process highly significant. For example, alpha-lipoic acid can inhibit EMT by suppressing the TGF-β signaling pathway and blocking the nuclear translocation of NF-κB, thereby increasing the radiosensitivity of tumor cells (72). Additionally, the BCSC-like cells formed during TGFβ-induced EMT can further give rise to cell populations with radioresistance (73). The interplay among EMT, BCSCs’ properties, and dysregulated oncogenic metabolism has been shown to be closely linked to radioresistance (74). Multiple studies suggest that targeting RT-induced EMT could improve RT outcomes by suppressing the reactivation of dormant hypoxic BCSCs, enhancing anti-tumor immune responses, modulating the expansion of radioresistant BCSCs, and optimizing the immune-mediated effects of RT (75).

While EMT-driven stemness acquisition is robustly supported by preclinical radiation models (70, 73, 74), clinical evidence for this process in human BC specimens post-RT remains indirect and largely observational. Small-molecule inhibitors targeting TGF-β or NF-κB have shown potential to reverse EMT *in vitro* (72), but their clinical utility in enhancing RT response remains under early-phase investigation (76, 77). The translational gap from preclinical EMT mechanisms to clinical radiosensitization strategies remains wide.

Controversies in EMT-driven radioresistance. While EMT-driven stemness acquisition is robustly supported by preclinical radiation models (70, 73, 74), clinical evidence for this process in human BC specimens post-RT remains indirect and largely observational. Small-molecule inhibitors targeting TGF-β (e.g., vactosertib, galunisertib) or NF-κB have shown EMT-reversing potential *in vitro* (72), but their clinical utility in enhancing RT response is limited to early-phase trials with radiological rather than BCSCs-specific endpoints. Several unresolved controversies contribute to this translational gap. First, the causal relationship between EMT and stemness acquisition is not invariant: while TGF-β-induced EMT generates BCSC-like cells with radioresistance in preclinical models, the extent to which RT-induced EMT in human tumors produces functionally equivalent stem cell populations remains uncertain. Second, EMT transcription factors exhibit non-redundant, context-dependent functions; for instance, Snail-driven EMT may confer different radioresistance profiles than those conferred by ZEB1- or TWIST1-mediated programs, yet most preclinical studies have not dissected these distinctions. Third, the clinical evidence for RT-induced EMT in human BC is largely observational and derived from small biopsy cohorts with limited temporal sampling; no prospective study has serially tracked EMT marker dynamics in matched pre- and post-RT tumor specimens. These limitations underscore the need for biomarker-driven trials that explicitly measure the burden of BCSCs and EMT status as co-primary endpoints.

## 3 TME and radioresistance

### 3.1 Components of the TME in BC

The TME consists of CAFs, the ECM, vascular network, proteoglycans, and a variety of immune cells. Among them, CAFs are closely associated with tumor tissues and play a key role in driving cancer progression and drug resistance by secreting growth factors and chemokines and by regulating the TME to recruit immune cells, thereby promoting tumor growth and survival (78, 79).

**Figure 1:**
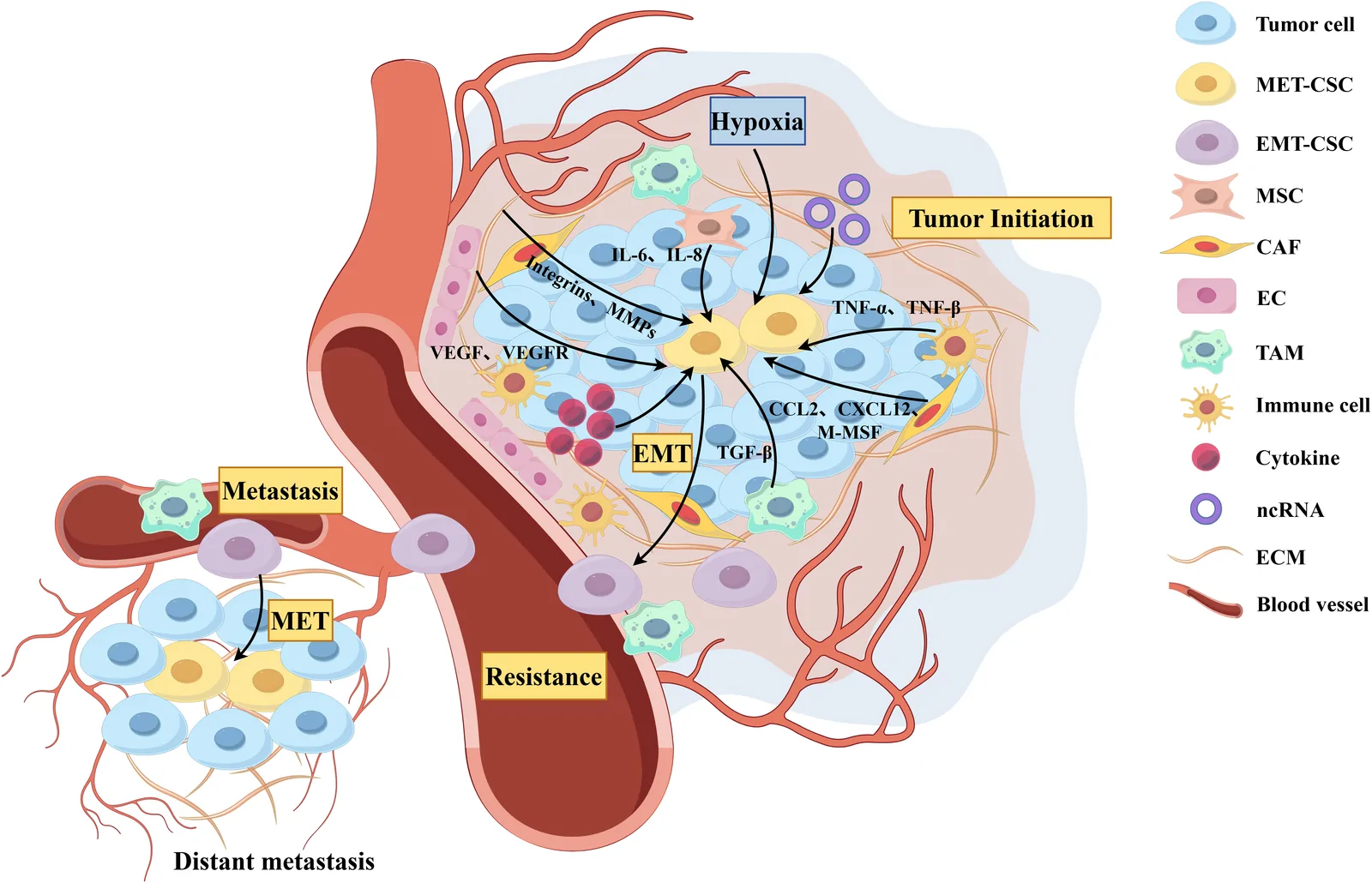
The biological characteristics and regulatory mechanisms of BCSCs. This schematic illustration depicts the dynamic interactions between BCSCs and the surrounding TME, which not only regulate the malignant phenotype of BC cells but also contribute to tumorigenesis, EMT, metastatic progression, and the establishment of a tumor-promoting microenvironment.

BC cells interact with a variety of immune cells to influence tumor growth, progression, malignancy, and treatment response (80, 81). The major immune components of BC TME include TAMs, neutrophils, natural killer (NK) cells, tumor-associated dendritic cells (TADCs), MDSCs, innate lymphoid cells (ILCs), and lymphocytes. These cells have the dual function of impeding tumor progression and promoting immunosuppression and tumor proliferation (82) (**Figure 1**).

The immunosuppressive landscape of the BC TME is well-characterized at both preclinical and clinical levels. Preclinically, studies have delineated the molecular crosstalk between BCSCs and immune cells, including PD-L1-mediated T-cell exhaustion, MDSC-induced stemness via IL-6/STAT3 signaling, and TAM-driven maintenance of BCSCs’ self-renewal through the Src/NF-κB axis (83–87). Clinically, this mechanistic understanding is anchored by the FDA approval of ICIs, such as pembrolizumab for TNBC (KEYNOTE-522), which represents the most advanced clinical application of TME-targeted therapy in BC (88, 89). However, the integration of RT with ICIs remains under active clinical investigation, with the optimal sequencing, dosing, and fractionation not yet established (90). Furthermore, the immunosuppressive microenvironment in most BC subtypes—particularly hormone receptor-positive tumors—limits the efficacy of ICIs, creating a “cold tumor” phenotype that constrains clinical benefits (91).

#### 3.1.1 Subtype-specific immune infiltration characteristics of the TME

The immune composition of BC TME demonstrates significant subtype specificity. Overall, TNBC and HER2+ subtypes exhibit higher immune cell infiltration than ER+ subtypes, but the nature of their immune landscapes differs fundamentally (5). TNBC is characterized by high levels of tumor-infiltrating lymphocytes (TILs), particularly CD8^+^ T cells and B cells, which are associated with its higher tumor mutational burden (TMB) and capacity for neoantigen generation, making it an ideal candidate for ICIs in combination with RT (4). However, TNBC concurrently harbors the highest MDSC infiltration levels; these cells are efficiently recruited to the TME via the CXCR2 axis (including CXCL8/IL-8, CXCL1, CXCL2, and CXCL5) and establish potent immunosuppression through ARG1, ROS, and PD-L1 expression.

In HER2+ subtypes, MDSC levels are intermediate, but HER2+ tumor cell-secreted TGF-β and CCL22 can recruit Tregs and MDSCs into the TME. Additionally, high M2-type CD163^+^ TAM counts correlate with poor prognosis in HER2+ BC patients, regardless of ER/PR status. Luminal A exhibits the lowest immune cell infiltration and MDSC levels, representing a typical ‘cold tumor’ that limits the efficacy of ICIs and explains the poor response to RT-immunotherapy combinations in this subtype. Luminal B can induce MDSC expansion under endocrine therapy or chemotherapy pressure, with stronger immunosuppressive function than Luminal A (5).

This subtype-specific immune landscape directly impacts RT-ICIs combination strategies: TNBC, with its ‘hot tumor’ characteristics, is the primary beneficiary population for RT-ICIs combinations (e.g., KEYNOTE-522 trial), whereas Luminal-type disease requires additional immune activation strategies (such as optimized RT fractionation, vaccination, or cytokine therapy) to overcome its intrinsic immune ‘cold’ state.

### 3.2 Mechanisms of CAFs enhancing radioresistance

CAFs are the main regulators of the ECM. They can regulate the release of certain growth factors associated with collagen deposition (e.g., type I collagen, fibronectin, and hyaluronic acid), leading to excessive deposition and linearization of the ECM. The dense, hardened ECM not only provides physical support for tumor cells but also impedes angiogenesis and oxygen diffusion by increasing interstitial tissue pressure, thereby maintaining a chronically hypoxic microenvironment within the tumor. The dense and hypoxic ECM generated by CAFs promotes radioresistance through the oxygen fixation hypothesis, whereby reduced oxygen tension favors the accumulation of repairable DNA radical intermediates over irreversible peroxide fixation (92), while also upregulating angiogenic factors and inducing the NRF2/HIF-1α antioxidant axis (Section 2.2.2) and metabolic reprogramming that collectively enhance cancer cell survival and adaptation (76, 93). Recent studies have shown that matrix hardness can also directly upregulate the expression of DNA repair genes (such as BRCA1, RAD51) and stem cell-related factors by activating the Hippo pathway effector molecules YAP/TAZ (94). Although RT can temporarily ablate some CAFs, the surviving CAFs often undergo senescence transformation, and their senescence-associated secretory phenotype (SASP) is associated with tumor growth, EMT, and radioresistance (95). SASP can express various proliferation-inducing factors (such as HGF, CXCL12, and EGF), which can induce treatment resistance by promoting tumor cell EMT, or support the progression and metastasis of tumor cells by upregulating glycolysis and ketone body production and metabolism (96, 97).

CAFs can also continuously engage in bidirectional communication with BC cells by secreting various cytokines, growth factors, and chemokines. Among them, TGF-β and hepatocyte growth factor (HGF) are two key mediators. TGF-β not only promotes the activation of CAFs and the secretion of ECM, but also activates the DDR pathway in cancer cells through Smad and non-Smad pathways (such as PI3K/Akt and MAPK), thereby enhancing NHEJ and HR repair capabilities (77). HGF binds to the c-Met receptor on cancer cells, strongly activating the downstream Akt and ERK1/2 signaling pathways and directly inhibiting radiation-induced apoptosis (98).

In addition, there is a significant “metabolic coupling” relationship between CAFs and BCSCs. The “oxidative glycolysis” effect (Warburg effect) of CAFs usually intensifies, generating large amounts of lactic acid, which is transported to cancer cells via monocarboxylate transporters (MCTs). The cancer cells then use this lactic acid as an efficient substrate for oxidative phosphorylation, generating abundant adenosine triphosphate (ATP), which supplies energy to the high-energy DDR pathway following radiation damage. This is the “reverse Warburg effect” (RWE) (92). Furthermore, CAFs directly assist cancer cells in scavenging RT-induced ROS by secreting antioxidants such as glutathione and ascorbic acid, thereby attenuating the indirect cytotoxicity of RT (Section 2.2.2) (99).

As mentioned earlier, RT itself is highly lethal to differentiated cells, but it may selectively enrich BCSCs with stronger DNA repair capacity and self-renewal potential. CAFs are crucial in inducing and maintaining the phenotype of BCSCs. The inflammatory factors they secrete, such as interleukin-6 (IL-6) and prostaglandin E2 (PGE2), can activate key stem cell-related pathways in cancer cells, conferring stem cell characteristics (100). These BCSCs are usually in a relatively quiescent state, possess stronger antioxidant capabilities and more effective activation of DNA damage checkpoints, and are the root cause of tumor recurrence and regeneration after RT (101). Furthermore, the CD10+GPR77+ CAFs subgroup has been confirmed to maintain BCSCs’ chemotherapy resistance by continuously activating the NF-κB signaling pathway. This mechanism is also applicable to the radioresistance scenario (102).

While RT not only directly kills local tumor cells but also induces immune-induced cell death (ICD) to release tumor-associated antigens—thereby activating systemic anti-tumor immune responses—preclinical co-culture and xenograft models have firmly established CAFs-derived paracrine signals (TGF-β, HGF, IL-6) as drivers of BCSCs radioresistance, with mechanisms including ECM remodeling, hypoxia maintenance, and SASP (94, 98, 100, 102). However, clinical validation of these findings remains sparse. Most evidence for CAFs-mediated hypoxia and ECM stiffness derives from animal studies (92). While FAP-targeted PET/CT imaging has entered clinical diagnostics for BC (103), CAFs-directed therapeutic strategies—including FAP-CAR-T, FAP mRNA vaccines, and TGF-β/PDGFR inhibitors—remain largely in phase I/II trials across solid tumors, with BC-specific efficacy data still accumulating (95, 104). The translation of preclinically validated CAFs-targeting approaches into clinically available radiosensitizers represents an urgent unmet need.

Metabolic coupling between CAFs and cancer cells also exhibits subtype specificity. In TNBC, CAFs produce substantial amounts of lactate via the “reverse Warburg effect” (RWE), which is transported to cancer cells via MCT1/MCT4 to drive oxidative phosphorylation and support high-energy-demand DNA damage repair. High CAFs-GPR34 expression in TNBC serves as a prognostic biomarker for immunotherapy response. In HER2+ BC, CAFs-secreted HGF activates Akt and ERK1/2 signaling through the c-Met receptor, directly inhibiting radiation-induced apoptosis—a mechanism particularly prominent in HER2+ cell lines (4). Luminal-type BC CAFs primarily promote ECM deposition and EMT through the TGF-β/Smad pathway rather than direct metabolic support. Therefore, CAF-targeting strategies must consider subtype differences: MCT1 inhibitors (e.g., AZD3965) have entered Phase I clinical trials in TNBC (105), whereas TGF-β inhibitors may be of greater value in Luminal-type disease (106, 107).

It is critical to emphasize that most mechanistic insights into CAFs-mediated radioresistance derive from preclinical co-culture and xenograft models, with limited direct validation in human BC specimens post-RT. While FAP-targeted PET/CT imaging has entered clinical diagnostics for BC, CAFs-directed therapeutic strategies, including FAP-CAR-T, FAP mRNA vaccines, and TGF-β/PDGFR inhibitors, remain largely in Phase I/II trials across solid tumors, with BC-specific efficacy data still accumulating. The translation of preclinically validated CAFs-targeting approaches into clinically available radiosensitizers, therefore, represents an urgent unmet need. Furthermore, metabolic coupling between CAFs and cancer cells exhibits subtype specificity: in TNBC, CAFs produce substantial l lactate via the RWE, transported via MCT1/MCT4, to drive oxidative phosphorylation in cancer cells, whereas Luminal-type BC CAFs primarily promote ECM deposition and EMT through TGF-β/Smad signaling rather than direct metabolic support. These distinctions imply that CAF-targeting strategies must be tailored to molecular subtype, with MCT1 inhibitors (e.g., AZD3965, currently in Phase I trials) holding greater potential in TNBC and TGF-β inhibitors potentially more valuable in Luminal-type disease.

## 4 Synergistic mechanisms of BCSCs and TME

The radioresistance of BC is not driven solely by BCSCs or the TME, but is the result of their combined action. BCSCs exhibit radioresistance due to their inherent DNA damage repair capacity, metabolic plasticity, and immune evasion; the TME further promotes radioresistance through mechanisms such as hypoxia, immunosuppression, and paracrine signaling by stromal cells. More importantly, there is a dynamic bidirectional regulation between BCSCs and the TME, forming a positive feedback loop of “microenvironment remodeling - stem cell maintenance” that jointly promotes radioresistance and tumor recurrence.

### 4.1 The synergistic effect of hypoxic microenvironment and BCSCs

Hypoxia serves as the core hub linking the TME and BCSC radioresistance. The dense ECM deposited by CAFs in the TME increases interstitial pressure, thereby hindering angiogenesis and oxygen diffusion, creating a chronic hypoxic microenvironment (92). In this environment, HIF-1α is stably expressed in BCSCs and promotes radioresistance through the following mechanisms: activating the Wnt/β-catenin and Notch signaling pathways to maintain self-renewal (Section 2.2.2) (51); switching to glycolysis and serine synthesis metabolic pathways to support the generation of antioxidants (55); upregulating antioxidant enzymes to eliminate reactive oxygen species (ROS) induced by radiation (52, 53). T; and the HIF-1α/NRF2 positive feedback loop to maintain the homeostasis of reactive oxygen species (54). Additionally, CAFs generate lactic acid via the RWE, which enters BCSCs via MCTs to fuel oxidative phosphorylation and supply ATP for the energy-demanding DDR pathway after RT (92).

### 4.2 The synergistic interaction between stromal cells and BCSCs

CAFs-BCSCs metabolic coupling operates bidirectionally: As described in Section 3.2, CAFs supply lactate via the RWE to fuel BCSCs’ oxidative phosphorylation, while BCSCs generate NADPH to support antioxidant systems (50). Beyond metabolism, IL-6 and PGE2 from CAFs activate stemness pathways in BCSCs (100), and the CD10^+^GPR77^+^ CAFs subset maintains BCSCs’ radioresistance through constitutive NF-κB activation (102).

After RT, TAMs polarize to the M2 type, secreting IL-10 and TGF-β to inhibit anti-tumor immunity while promoting BCSCs’ self-renewal. These M2-type macrophages further recruit additional M2 subsets via CCL2/CXCL1 and interact with BCSCs to activate Src and NF-κB signaling, thereby driving the release of IL-6 and TNF-α to maintain BCSCs’ stemness. Concurrently, the dense ECM deposited by CAFs directly upregulates DNA repair genes (BRCA1, RAD51) and stemness-related factors by activating the Hippo pathway effectors YAP/TAZ. After RT, the surviving CAFs undergo senescence transformation, expressing proliferation-inducing factors such as HGF, CXCL12, and EGF in their SASP, thereby inducing tumor cell EMT or upregulating glycolysis and ketone body production, promoting the survival and radioresistance of BCSCs.

### 4.3 Remodeling of the immune microenvironment and the escape of BCSCs

Tumor-infiltrating lymphocytes (TILs) contain mainly CD8^+^ T cells, CD4^+^ T cells, and Tregs. Interactions between these T cell subsets and CSCs are critical for mediating immune escape, therapeutic resistance, and maintenance of stem cell identity. As major cytotoxic effector cells, CD8^+^ T cells clear tumor cells by secreting perforin, granzymes, and other cytotoxic molecules (108). However, CSCs can evade CD8^+^ T cell-mediated killing by a variety of mechanisms. In BC, BCSCs significantly upregulate the immune checkpoint ligand PD-L1. PD-L1 not only activates the PI3K/Akt and ERK1/2 pro-survival pathway, but also blocks ZAP70 phosphorylation downstream of the TCR by binding to PD-1 on the surface of CD8^+^ T cells, inhibits the release of perforin, granzyme B, and IFN-γ, and induces T cells to undergo mitochondria-dependent apoptosis (83). Therefore, the PD-1/PD-L1 signaling axis forms a bidirectional positive feedback loop between BCSCs and immune cells: it protects BCSCs from immune surveillance and creates an immunosuppressive microenvironment, thereby providing a cellular and molecular basis for tumor progression, metastatic dissemination, and resistance to RT (84).

BCSCs remodel the TME to maintain their stemness through paracrine mechanisms: first, BCSCs induce TADCs to highly express CCL2, a chemokine that directly enhances the self-renewal ability of BCSCs (85); second, MDSCs remotely regulate the BCSCs’ phenotype with the help of the IL-6/STAT3 and NO/Notch signaling pathways (86); and then, the TAMs interact with BCSCs to promote the activation of Src and NF-κB signaling pathways, drive the release of cytokines such as IL-6 and TNF-α, and maintain BCSCs’ stemness (87). Meanwhile, TME-enriched IL-4, IL-10, and TGF-β reprogrammed monocytes and macrophages to an M2-like phenotype; M2-TAMs not only inhibited CD8^+^ T-cell function and recruited Tregs but also further recruited M2 subpopulations through the secretion of CCL2 and CXCL1. M2-TAMs, in turn, reversed the release of TGF-β and IL-10, reinforcing the DNA repair and ROS-scavenging capacity of BCSCs, culminating in a positive feedback loop of immunosuppression-stem maintenance (109–111).

PD-L1-mediated immune evasion varies across subtypes. In TNBC, BCSCs significantly upregulate PD-L1 expression, not only activating PI3K/Akt and ERK1/2 pro-survival pathways but also inducing mitochondrial-dependent apoptosis in CD8^+^ T cells by blocking ZAP70 phosphorylation downstream of the TCR. This mechanism creates a paradox in TNBC: despite abundant immune cells, activation of the PD-L1/PD-1 axis induces functional exhaustion. Pembrolizumab combined with chemotherapy in the KEYNOTE-522 trial significantly improved the pathological complete response (pCR) rate in early TNBC, establishing ICIs as standard therapy for this subtype (89). However, in Luminal-type BC, the scarcity of TILs and low PD-L1 expression limit the efficacy of ICI monotherapy, and RT-induced immunogenic cell death (ICD) may be insufficient to trigger effective systemic immune responses. In HER2+ subtypes, HER2 signaling itself upregulates PD-L1 expression, while anti-HER2 therapy (e.g., trastuzumab) can reduce PD-L1 levels by blocking the HER2-PI3K-Akt pathway, thereby enhancing RT-induced immune activation (112).

### 4.4 The cross-collaborative effect of signaling pathways

PI3K/Akt/mTOR Pathway: As the core integrator of microenvironmental signals (Sections 2.2.2 and 3.2), this pathway promotes cell cycle arrest at the radioresistant G2/M phase, enhances ATM-Chk2-mediated DDR, and inhibits apoptosis, with inputs from CAF-derived growth factors, PD-L1-mediated signaling, and HIF-1α-driven mTOR activation under hypoxia.

Notch-Wnt-EGFR Pathway: The key pathway for maintaining stem cell characteristics. TME factors induce the expression of Syndecan-1 and simultaneously activate three pathways: IL-6/STAT3, Notch, and EGFR, working together to maintain the BCSCs phenotype. HIF-2α acts as a ‘molecular switch’ in hypoxic TME to initiate Wnt and Notch pathways (51), synergizing with the HIF-1α/NRF2 axis (Section 2.2.2). RT-induced EMT activates the Notch and NF-κB pathways and upregulates EMT transcription factors such as Snail, allowing non-stem cells to acquire BCSCs’ characteristics, further enriching the population of radioresistant cells.

Coordinated Regulation of Cell Cycle Checkpoints: Due to the dysfunction of the p53/p21 signaling axis in BCSCs, they rely more on the G2/M checkpoint for DNA repair. Surviving BCSCs after RT screening finely regulate key cell cycle regulatory factors such as Wee1 kinase, CDC25 family members, and PLK1, overcoming G2/M checkpoint blockage and re-entering the cell cycle. These cells tend to converge towards the S phase, leveraging the HRR advantage of S-phase cells to repair radiation-induced DSBs with high accuracy, thereby maintaining genomic stability and self-renewal capacity.

### 4.5 The vicious cycle of TME-EMT-immune escape dysregulation and radioresistance

In TME, TGF-β and IL-6 induce EMT, enabling tumor cells to acquire a BCSC phenotype (63, 65). BCSCs recruit Tregs and M2-type macrophages. TAMs further maintain stemness through the Src/NF-κB axis and secretion of TGF-β. The stemness phenotype is accompanied by enhanced DNA repair capacity, anti-apoptotic effects, and abnormal cell cycle regulation, leading to radioresistance. Radioresistant cells secrete more CCL2, VEGF, MMPs, etc., further deteriorating the microenvironment and forming a positive feedback loop of “immune suppression - stem cell maintenance – radioresistance”. Furthermore, CAFs can secrete TGF-β/HGF, thereby influencing the DDR and inhibiting apoptosis (Section 3.2) (77, 98).

Clarifying the molecular basis of the BCSCs-TME axis will provide a theoretical foundation for the simultaneous targeting of BCSCs and the TME, enabling the development of a new generation of combined therapeutic strategies and significantly increasing the overall efficacy of antitumor therapy.

### 4.6 Spatiotemporal dynamics of RT-induced TME remodeling and BCSCs plasticity

The radioresistance of BC is not merely a consequence of static molecular alterations but emerges from dynamic, time-dependent remodeling of the TME and reversible phenotypic transitions of BCSCs. Understanding these spatiotemporal dimensions is essential for optimizing the timing and sequencing of combination therapies.

#### 4.6.1 Temporal dynamics of TAM reprogramming following RT

RT triggers a biphasic temporal evolution of TAM populations that critically determines therapeutic outcomes. First, in the immediate phase (0–48 hours), direct radiation-induced cytotoxicity causes a transient depletion of intratumoral TAMs, coinciding with the release of damage-associated molecular patterns (DAMPs), such as HMGB1 and calreticulin, from dying tumor cells. This early window represents a potential “immune sensitization phase” during which the TME is transiently primed for immunostimulatory intervention. Second, in the early post-RT period (3–21 days), compensatory TAM influx occurs via CCL2-CCR2 and CSF1-CSF1R signaling axes, recruiting Ly6C^+^ inflammatory monocytes that differentiate into TAMs within the irradiated tissue. Notably, in cervical cancer patients undergoing radiochemotherapy, TAMs at 3 weeks post-treatment exhibited a hybrid M1/M2 phenotype—co-expressing both pro-inflammatory and immunosuppressive gene signatures—indicating functional plasticity rather than terminal polarization. This intermediate phase may represent a critical therapeutic window during which TAM repolarization strategies could be most effective. Third, in the late/recurrence phase (months to years), global TAM abundance often returns to pre-treatment levels but with altered spatial distribution and transcriptional programs. In glioblastoma—an important comparator for understanding TAM dynamics, though not directly generalizable to BC—recurrence is driven by lipid-laden macrophages accumulating in hypoxic, pseudopalisading regions, demonstrating that TAM spatial niches, not merely their numbers, dictate radioresistance. However, these temporal dynamics have been primarily characterized in cervical cancer and glioblastoma; their direct applicability to BC requires prospective validation in BC-specific cohorts with longitudinal sampling. Whether analogous spatial redistribution occurs in BC recurrence remains to be determined through longitudinal spatial transcriptomic studies. Direct extrapolation of these spatiotemporal dynamics from glioblastoma to BC is currently unsupported and requires prospective longitudinal studies in breast cancer patients.

#### 4.6.2 Spatial organization of BCSCs and TAMs within the TME

BCSCs are not randomly distributed but occupy distinct spatial niches governed by microenvironmental signaling gradients. Computational modeling and experimental validation in BC models have demonstrated that TGF-β diffusion from the tumor core, combined with Notch-JAG1 signaling, generates spatially segregated subpopulations of BCSCs: mesenchymal-like BCSCs localize to the invasive tumor edge, whereas hybrid epithelial/mesenchymal (E/M) BCSCs reside in the tumor interior. This spatial partitioning has direct implications for RT planning, as the invasive edge may receive sublethal radiation doses in partial breast irradiation protocols, potentially enriching for mesenchymal BCSCs with enhanced radioresistance.

TAMs exhibit complementary spatial patterning. In pembrolizumab-plus-RT responders across multiple solid tumors (including TNBC), antigen-presenting macrophages spatially co-localize with effector CD8^+^ T cells, forming immunostimulatory micro-niches. Conversely, in non-responders, CD163^+^ M2-TAMs congregate with SFRP2^+^ CAFs in perivascular regions, establishing an immunosuppressive barrier that shields tumor vasculature from immune attack. These spatial configurations suggest that RT field design and dose-painting strategies should account for TAM-BCSCs’ spatial co-localization patterns, though such precision targeting remains investigational.

#### 4.6.3 Dose-fractionation-dependent temporal windows

Radiation parameters exert non-overlapping immunomodulatory effects, creating distinct temporal windows for combination therapy. High-dose RT (HDR, >8–10 Gy per fraction) induces extensive necrosis and a cytokine storm, triggering a TAM influx that is subsequently dominated by immunosuppressive TGF-β and IL-10 signaling, thereby favoring M2 polarization. Moderate-dose radiation (2–8 Gy) creates a “golden window” for M1 polarization through balanced DAMP release and type I interferon production. Low-dose radiation (≤2 Gy) primarily functions as an “immune microenvironment modulator,” repolarizing existing TAMs without substantial recruitment.

Conventional fractionation (1.8–2 Gy×25–30 fractions) provides repeated, sustained immune stimulation through sequential antigen and DAMP release, enabling dynamic evolution of TAM polarization over the treatment course. Hypofractionation (e.g., 8 Gy×3 fractions) generates a potent “*in situ* vaccine” effect but simultaneously creates a highly immunosuppressive milieu. These dose-dependent temporal dynamics imply that the optimal sequencing of ICIs or TAM-targeting agents relative to RT may vary with fractionation scheme—a hypothesis that requires prospective clinical validation.

**Figure 2:**
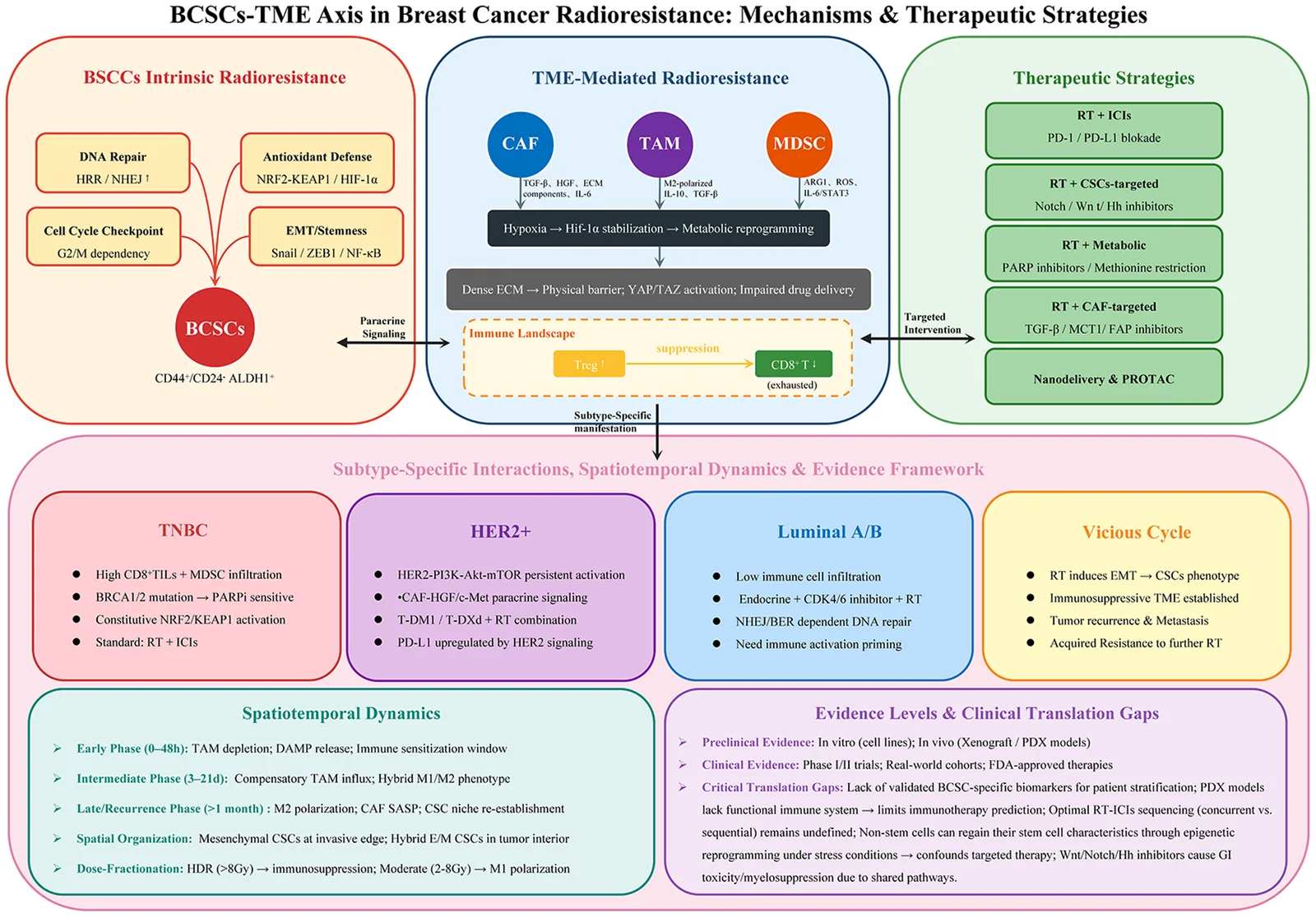
Integrated schematic of the BCSCs-TME axis in radioresistance. Upper panel: intrinsic BCSCs resistance mechanisms (DNA repair, NRF2/HIF-1α antioxidant defense, cell cycle dysregulation, and EMT-driven stemness acquisition) and TME-mediated protective networks (CAFs-ECM remodeling, metabolic coupling, hypoxia, and immunosuppression via TAMs/MDSCs/Tregs). Middle panel: emerging combination therapies (RT+ICIs, CSC-directed agents, metabolic interventions, CAFs-targeted therapies, and nanodelivery/PROTAC platforms). Lower panel: subtype-specific interaction networks (TNBC, HER2+, Luminal A/B), the vicious cycle of RT-induced EMT-immunosuppression-recurrence, spatiotemporal dynamics of RT-induced TME remodeling (early/intermediate/late phases, spatial niche organization, dose-fractionation effects), and the evidence-to-practice framework distinguishing preclinical data, clinical trials, and critical translation gaps.

Collectively, the BCSCs-TME axis establishes a robust network of intrinsic and extrinsic radioresistance through bidirectional paracrine signaling, spatial niche partitioning, and temporal evolution following RT (**Figure 2**). These insights provide the rationale for the multimodal therapeutic strategies discussed below.

## 5 Intervention strategies: targeting BCSCs and remodeling the TME

### 5.1 Targeted therapeutics for BCSCs: navigating heterogeneity and plasticity

The therapeutic strategies targeting BCSCs aim to eradicate tumor recurrence and treatment resistance. However, the two inherent characteristics of BCSCs - functional and phenotypic heterogeneity and dynamic plasticity - make the clinical translation of these strategies extremely complex (113, 114). Heterogeneity is manifested as distinct BCSC subpopulations, which may rely on different signaling pathways for self-renewal and survival (115). This implies that targeting a single marker or pathway may eliminate only a portion of BCSCs, thereby allowing the emergence of drug-resistant clones that drive recurrence. What is even more challenging is that BCSCs exhibit remarkable plasticity, enabling them to reversibly switch between stem cell-like and non-stem cell states (116) and to adjust their phenotypes in response to therapeutic stress (117) or microenvironmental signals (118).

Therefore, effective surface markers or signal-dependent characteristics before treatment may be downregulated or lose their efficacy during treatment, leading to adaptive escape (119). In view of this, effective BCSCs targeted therapeutic strategies must go beyond the traditional approach of targeting a single static target and shift to one of the following two approaches: One is to develop combined therapies that simultaneously inhibit multiple core pathways that maintain stem cell characteristics to prevent compensatory activation (117, 120); the other is to develop strategies that can directly intervene in the plasticity of stem cells, targeting and regulating the key transcriptional or epigenetic hubs that control their state transitions (121, 122). In the following text, within this framework, the current targeted drugs targeting each pathway will be systematically reviewed. We will discuss both the therapeutic potential of these agents and the limitations imposed by the dynamic evolution of their targets within the context of BCSCs’ heterogeneity.

Targeting key signaling pathways that regulate cancer stem cell self-renewal, proliferation, and differentiation has become a highly promising strategy in tumor therapy. Evidence suggests that inhibition of core pathways such as Notch, Wnt, and Hedgehog (Hh) significantly impairs the self-renewal capacity of BCSCs (123, 124).

Notch signaling is decisive for the homeostatic maintenance of BCSCs, and its aberrant activation is closely associated with radioresistance in BC cells (125, 126). Gamma-secretase inhibitors (GSIs) block the release of the Notch intracellular domain (NICD), thereby interrupting the signaling cascade. Phase I clinical trials have shown that GSI MK-0752, in combination with docetaxel, significantly reduces the ratio of BCSCs and inhibits microsphere formation in primary tumors (127, 128).

Wnt/β-catenin activation significantly enhances self-renewal and drug resistance in BCSCs (129). Small molecule inhibitors or neutralizing antibodies inhibit BCSCs proliferation both *in vitro* and in xenograft models (130, 131). Vantictumab, a monoclonal antibody in phase I clinical trials, reduces the proportion of BCSCs and promotes cell differentiation in PDX models by blocking β-catenin nuclear translocation through inhibition of the Wnt receptor. Inhibition of WNT1 induced phenotypic transformation of BCSCs, reducing their tumorigenic potential and migratory capacity, and protein kinase D1 inhibitors attenuated stem cell properties and restored chemosensitivity via the GSK3-β-catenin axis (132, 133). In addition, the HSP90 inhibitor NVPAUY922 downregulates the expression of the proliferating cell nuclear antigen (PCNA)-related factor RAF, thereby inhibiting BCSCs’ self-renewal and intratumor heterogeneity (134).

The Hh signaling pathway maintains ‘BCSCs’ stemness and improves interactions between tumors and the stroma in a paracrine/autocrine manner, thereby driving disease progression (135). Cyclobenzaprine, a smooth muscle inhibitor, dramatically increases the responsiveness of BCSCs to paclitaxel by blocking the Hh cascade. A phase II clinical trial of the Hh inhibitor GDC-0449 in combination with paclitaxel, epirubicin, and cyclophosphamide in TNBC has been completed to assess efficacy, suggesting the viability and safety of Hh-targeted therapy (136). Other agents evaluated in early-phase trials include the gamma-secretase inhibitor RO4929097 (NCT01149356) (137) and the Wnt inhibitor vantictumab (NCT01973309) (138), though their development has been limited by gastrointestinal toxicity and bone-related adverse events, respectively.

### 5.2 Immunomodulatory therapy

Although the immune system can eliminate cancer cells, malignant tumors evade immune detection through various mechanisms. Cancer cells alter the TME by modulating signaling, reducing antigen-presentation efficiency, and inducing T-cell depletion, among other mechanisms, thereby establishing an immunosuppressive microenvironment (139).

ICIs, including PD-1/PD-L1 (140) and CTLA-4 inhibitors, restore T-cell-mediated anti-tumor activity and are widely used in BC management (88, 140–143). Although ICIs have shown clear efficacy in improving clinical outcomes in BC patients, primary resistance still occurs in a significant proportion of cases, and the mechanism is partly attributed to immune escape due to impaired antigen presentation and abnormal activation of signaling pathways (144).

Chimeric antigen receptor T-cell (CAR-T) therapy has achieved breakthroughs in hematologic malignancies (145), but its application in solid tumors such as BC is still in the exploratory stage. To date, phase I clinical trials show that the therapy has a manageable safety profile in BC patients, and disease stabilization can be achieved in some patients (146–149). The principle of this technology is to genetically modify T cells to express chimeric antigen receptors that recognize tumor-associated antigens. In BC (especially TNBC), potential CAR-T targets include HER2, MUC1, EGFR, MSLN, and nucleolin (150, 151). Among them, HER2, as a member of the EGFR family, can activate oncogenic signaling pathways by forming heterodimers with HER3 and HER4. Preclinical studies have shown that CAR-T therapies targeting HER2/HER3/HER4 can effectively inhibit tumor proliferation and retard tumor growth (152, 153). However, CAR-T efficacy in solid tumors is still hampered by a variety of factors, including antigenic heterogeneity, T-cell depletion, poor tumor infiltration, and suppression of effector activity by an immunosuppressive TME (154–156).

### 5.3 Synergistic strategies for BC RT

RT is the cornerstone of radical strategies for BC, combining the dual benefits of local-regional control and distant metastasis prevention; however, residual BCSCs, with their highly efficient DNA repair and immune-escape mechanisms, have become the primary cytological basis for recurrence and drug resistance. The integration of RT with immunotherapy or BCSCs-targeted drugs has become a hotspot for breaking the efficacy bottleneck.

#### 5.3.1 RT combined with ICIs

Preclinically, RT combined with ICIs has demonstrated synergistic anti-tumor immunity and abscopal effects in BC models, with mechanisms including ICD-induced antigen release, CXCL16-CXCR6-mediated T-cell recruitment, and IFN-γ-driven oxidative stress in BCSCs (157–162). These foundational studies provided the rationale for clinical translation. To date, clinical evidence is primarily derived from phase I/II trials and retrospective cohorts. The P-RAD trial (NCT04443348) is actively evaluating concurrent versus sequential RT plus pembrolizumab (90). Real-world safety data have confirmed feasibility but also highlighted an increase in grade 1 radiation dermatitis (83.3% vs 43.9% in controls) (163), underscoring that safety profiles observed preclinically may not fully predict clinical toxicity. Optimal RT dose and fractionation for immune activation (8–10 Gy × 3 fractions from preclinical studies (164); alternative regimens such as 7.5 Gy × 1 or 2 Gy × 5 in clinical trials (165)) remain to be standardized.

RT can remodel the tumor immune microenvironment through multiple mechanisms: (1) upregulation of mutational load and genotoxic stress, leading to the release of tumor-specific neoantigens; (2) induction of proinflammatory cytokine secretion; and (3) enhancement of intravascular adhesion molecules, which synergistically increase the infiltration of CD8^+^ T-cells and dendritic cells (DCs) (166). As a result, the irradiated tumor can be transformed into a “vaccine in situ”: apoptotic/necrotic tumor cells are taken up by DCs, which efficiently activate tumor-specific CD8^+^ T cells via the MHC-I cross-presentation pathway, forming systemic immune memory. And sequential or simultaneous application of RT with ICIs can further amplify the above effects and reverse acquired ICIs resistance in multiple models (167). In an *in situ* model of BC, RT combined with ICI blockade significantly upregulated intratumoral CD8^+^ T-cell infiltration, decreased the proportion of MDSCs, and inhibited tumor growth after rechallenge (157). In TNBC models, RT combined with ICIs significantly reduced ALDH^+^ BCSCs and delayed local recurrence (158).

RT upregulates CXCL16 expression in mouse BC cells and recruits CXCR6^+^ T cells into the TME via the CXCL16-CXCR6 axis. In a 4T1 mouse model, RT combined with ICI blockade not only inhibited primary tumor growth but also significantly reduced lung metastasis and prolonged OS (159, 160). Further mechanistic studies showed that DNA damage induced by RT synergized with the massive secretion of IFN-γ by CD8+ T cells after ICIs, thereby inducing lethal oxidative stress in BCSCs (161). Several studies have demonstrated that the combined therapy enhances CD8+ T-cell infiltration and amplifies the killing effect on BCSCs through IFN-γ-dependent DDR (158, 162).

The clinical evidence and applicability of RT-ICIs combinations vary significantly across subtypes.

##### 5.3.1.1 Clinical toxicity profile and monitoring considerations

The safety profile of RT-ICIs combinations in BC requires careful prospective evaluation. In a retrospective real-world cohort of 89 TNBC patients receiving concurrent pembrolizumab and adjuvant RT, El Fakih et al. reported a significantly higher rate of Grade 1 radiation dermatitis (83.3% vs. 43.9%) (168). Long-term toxicity concerns include: (i) radiation pneumonitis and cardiotoxicity, with median follow-up of only 16 months insufficient for detection; (ii) pembrolizumab-associated myocarditis (incidence <1% but fatal), with unknown synergistic risk when combined with thoracic RT; and (iii) a global meta-analysis showing higher cardiac adverse event risk in non-lung non-melanoma cancers (RR = 1.42, 95% CI 1.21–1.65), suggesting BC patients may be particularly vulnerable. Mechanisms underlying RT-ICIs synergy are described in Section 5.3.1.

##### 5.3.1.2 Trial design challenges and biomarker gaps

According to consensus recommendations from NRG Oncology, ASTRO, ACR, and allied organizations, early-phase drug-RT trials face unique challenges distinct from drug-only development: (i) dose-limiting toxicity (DLT) windows must extend beyond conventional 28-day cycles to encompass the full RT course and late toxicity monitoring; (ii) efficacy endpoints requiring years of follow-up (e.g., locoregional recurrence, OS) necessitate development of early surrogate markers such as ctDNA clearance or functional imaging response; (iii) the lack of validated biomarkers to direct patient selection for RT-ICIs combinations remains a critical barrier—unlike melanoma or NSCLC, where PD-L1 and TMB are established predictive markers, their utility in BC (particularly when combined with RT) is unclear and heterogeneous. These structural limitations of clinical trial design partially explain why preclinically promising RT-ICIs combinations have not yet yielded definitive Phase III evidence in BC.

TNBC is the primary beneficiary population for this combination strategy. The KEYNOTE-522 trial established pembrolizumab plus chemotherapy as standard neoadjuvant therapy for early TNBC, while the ongoing P-RAD trial (NCT04443348) is evaluating concurrent versus sequential RT plus pembrolizumab (169). Real-world data show that TNBC patients receiving RT-pembrolizumab combination experienced significantly higher Grade 1 radiation dermatitis (83.3% vs. 43.9%), but long-term cardiopulmonary toxicity requires longer follow-up. In the 4T1 TNBC mouse model, RT combined with anti-PD-1 therapy not only inhibited primary tumor growth but also significantly reduced lung metastasis and prolonged OS, involving CXCL16-CXCR6 axis-mediated T-cell recruitment and IFN-γ-driven oxidative stress killing of BCSCs.

In HER2+ BC, RT combined with anti-HER2 therapy (trastuzumab, pertuzumab, or T-DM1) shows synergistic potential. The KATHERINE trial confirmed that T-DM1 as adjuvant therapy for residual disease after neoadjuvant treatment significantly improved IDFS and OS. T-DM1 combined with RT is generally safe in HER2+ BC, though vigilance for specific severe toxicities is warranted (170). Preliminary results from the DESTINY-Breast05 trial indicate that T-DXd further improved IDFS in high-risk HER2+ patients compared to T-DM1, suggesting next-generation ADC-RT combinations may represent a future direction. Additionally, PIK3CA mutations in HER2+ BC correlate with resistance to anti-HER2 therapy; combining PI3K inhibitors (e.g., alpelisib) with RT may overcome this mechanism of resistance (171, 172).

In Luminal-type BC, RT combined with CDK4/6 inhibitors (e.g., palbociclib) and endocrine therapy is being actively investigated. The PATINA trial evaluated palbociclib combined with anti-HER2 therapy and endocrine therapy in HR+/HER2+ metastatic BC. For HR+/HER2- subtypes, RT combined with endocrine therapy and CDK4/6 inhibitors may enhance radiosensitivity by inducing cell-cycle arrest, though cumulative hematologic toxicity warrants attention. PARP inhibitors in BRCA-mutated Luminal-type disease (OlympiA trial) also provide a new radiosensitization option.

Fundamental evidence-to-practice gaps. A fundamental limitation in the current literature is the profound discordance between the abundance of preclinical mechanistic data and the scarcity of high-level clinical evidence. Many pathways mechanistically validated in BCSC models, such as the NRF2-KEAP1 antioxidant axis, HIF-1α-mediated metabolic reprogramming, and CAFs-ECM crosstalk, lack corresponding clinical biomarkers or approved targeted therapies to address radioresistance. Conversely, while clinical trials have established the efficacy of ICIs and PARP inhibitors in BC, the specific contribution of these agents to reversing BCSCs-intrinsic radioresistance in patients remains inferred rather than directly proven. This evidence gap complicates the rational design of combination regimens. Future research must prioritize translating preclinically validated biomarkers into clinical companion diagnostics and designing biomarker-driven trials that explicitly distinguish BCSC-targeting effects from general tumor cytotoxicity.

### 5.3.2 RT combined with BCSCs-directed agents

Preclinical evidence strongly supports the combination of RT with BCSCs-directed agents. For example, all-trans retinoic acid (ATRA) inhibits Wnt/β-catenin signaling, reducing ALDH^+^ BCSCs by approximately 70% in TNBC models and significantly delaying tumor recurrence when combined with RT (173). Methionine restriction, synchronized with RT, selectively clears BCSCs, whereas PARP inhibitors primarily target proliferating tumor cells, achieving 80% tumor regression in PDX models (174). However, clinical evidence for these specific combinations remains scarce. Most available clinical data come from single-agent phase I/II trials evaluating BCSC-targeting agents alone, such as the Notch inhibitor MK-0752 (128), the Wnt inhibitor vantictumab (NCT01973309) (130), and the Hedgehog inhibitor vismodegib (136), which have shown limited efficacy due to toxicity and BCSCs’ plasticity (175, 176). The translation of preclinically successful RT plus BCSCs-targeted combinations into clinical protocols is a critical unmet need.

### 5.4 Emerging therapies

#### 5.4.1 Nanoparticle-based drug delivery systems

Nanocarriers have become an important drug delivery system in oncology to overcome the drawbacks of systemic toxicity, incremental dose requirements, and acquired resistance associated with conventional formulations (177). Its nanoscale size (10–200 nm) significantly improves drug bioavailability and enables precisely targeted delivery and controlled drug-release kinetics. It also enhances cellular uptake efficiency, thereby promoting the effective enrichment of therapeutic drugs in tumor tissues (178). By virtue of the high permeability of tumor vasculature and the absence of lymphatic return, nanoparticles can be used to achieve passive targeted aggregation and controlled release in the focal area via the enhanced permeation and retention (EPR) effect (179).

Preclinically, P-selectin-mediated nanodelivery systems have been used to co-load PARP inhibitors (niraparib) with PD-L1 inhibitors, thereby enhancing focal accumulation in BRCA1-deficient tumors. Radiation-induced upregulation of P-selectin promotes targeted delivery of such nanocarriers to metastatic foci, including brain metastases (157). PROTAC (proteolysis-targeting chimera) technology, based on the ubiquitin-proteasome system (UPS), has also been integrated into nanoplatforms: the checkpoint nanoprocessor PROTAC (NPRO) can significantly augment anti-tumor immune response by targeting SHP2 while concurrently inhibiting CD47/SIRPα and PD-1/PD-L1 (180); BRD4-targeted PROTAC modulates PD-L1 and c-Myc expression (181); and carbon-dot-based PD-L1-targeted PROTAC is under investigation (182).

Clinically, however, these nanodelivery modalities remain in nascent stages. A phase I trial (NCT03739931) evaluating mRNA lipid nanoparticles (encoding OX40L, IL-23, IL-36γ) in advanced solid tumors/lymphoma represents one of the first clinical steps (41). ER-targeting PROTACs are being evaluated in phase I trials for hormone receptor-positive BC (ASCO 2026). *In vivo* delivery, pharmacokinetics, long-term safety, and large-scale manufacturing remain unresolved barriers, with no approved nanodelivery-based radiosensitizer currently available for BC patients (177).

#### 5.4.2 CRISPR-Cas9 gene editing

The CRISPR-Cas9 system has driven a revolutionary breakthrough in genome-editing technology and has shown broad application prospects in tumor biology research (183). With the help of gene knockout, epigenetic modification, and targeted mutation, CRISPR-Cas9 can efficiently target oncogenes and significantly inhibit the proliferation and survival of cancer cells (184, 185).

Preclinically, knockdown of the chemokine receptors CXCR4 and CXCR7, alone or in combination, significantly impaired the migratory ability and *in vivo* growth rate of TNBC cells (186). Deletion of the E3 ubiquitin ligase UBR5 coding region can prevent TNBC from growing and spreading *in vivo (*187). Hyaluronic acid-modified core-shell nanoparticles co-delivering CRISPR/Cas9 and chemotherapy have been shown to downregulate FBXO44 expression, reverting BCSCs to normal tumor cells and effectively inhibiting TNBC growth (40). This system is also useful for studies of non-coding RNA loss of function.

Clinically, however, CRISPR-based therapies are still in preclinical/early clinical stages. While CRISPR-Cas9 screening has been used to identify prognostic signatures in TNBC, *in vivo* delivery, off-target effects, immunogenicity, and potential genotoxicity remain major barriers to clinical translation (184, 185). No CRISPR-based radiosensitizer has been approved for BC patients to date. Future directions include ex vivo editing to enhance CAR-T and the development of tissue-specific delivery vectors.

## 6 Challenges and future perspectives

### 6.1 Current research limitations

A fundamental limitation in the current literature is the profound discordance between the abundance of preclinical mechanistic data and the scarcity of high-level clinical evidence. Many pathways mechanistically validated in BCSC models—such as the NRF2-KEAP1 antioxidant axis, HIF-1α-mediated metabolic reprogramming, and CAFs-ECM crosstalk—lack corresponding clinical biomarkers or approved targeted therapies for radioresistance (47, 54, 92). Conversely, while clinical trials have established the efficacy of ICIs and PARP inhibitors in BC, the specific contribution of these agents to reversing BCSCs-intrinsic radioresistance in patients remains inferred rather than directly proven (39, 43). To provide a comprehensive overview of the molecular pathways, therapeutic targets, and the current status of clinical translation, we summarize the key mechanisms of BCSCs-intrinsic radioresistance and TME-mediated resistance in **Table 1**, with explicit distinction between preclinical and clinical evidence. This evidence gap complicates the rational design of combination regimens. Future research must prioritize translating preclinically validated biomarkers into clinical companion diagnostics and designing biomarker-driven trials that explicitly distinguish BCSC-targeting effects from general tumor cytotoxicity.

**Table 1:** Summary of key mechanisms, therapeutic targets, supporting evidence, and clinical status.

Category	Mechanism/pathway	Key molecular targets	Cellular/functional outcomes	Supporting evidence (preclinical)	Supporting evidence (clinical)	Clinical status
BCSCs Intrinsic Resistance	DNA damage repair	BRCA1, RAD51, ATM/ATR, CHK1/CHK2, CDK1/2, DNA-PKcs	Enhanced HRR and NHEJ; G2/M checkpoint dependency; efficient DSB repair; survival of BCSCs after IR	BCSCs exhibit higher expression of DNA repair-related genes than non-stem cells (31, 32); ATM/ATR phosphorylates CHK1/CHK2, inhibiting CDC25C to block G2/M checkpoint (36–38); BCSCs rely more on G2/M checkpoint due to p53/p21 dysfunction (35).	PARP inhibitors (olaparib, niraparib, talazoparib) approved for BRCA-mutated BC; olaparib + RT (NCT03109080); olaparib + durvalumab (MEDIOLA) (39).	Phase I/II ongoing; olaparib + RT in TNBC shows manageable toxicity and 3-year EFS 65%; niraparib + pembrolizumab (TOPACIO) ORR 21% overall, 47% in BRCA-mut (43).
	NRF2-KEAP1 axis	NRF2, KEAP1, HO-1, NQO1, GCLC, ZMYND8, GSTP1, GPX2, GCLM	ROS scavenging; GSH synthesis; ferroptosis resistance; maintains low ROS in BCSCs	NRF2 continuously activated in BCSCs; KEAP1 binding abnormal, NRF2 translocates to nucleus and upregulates HO-1, NQO1, GCLC (45–47); ZMYND8 acts as NRF2 co-activator (48); SOD2 and CAT at high levels (49); BCSCs enhance antioxidant state via metabolic reprogramming (OXPHOS, FAO) (50).	No BCSCs-specific NRF2 inhibitor approved; brusatol (NRF2 inhibitor) in preclinical development; NRF2 activation correlates with poor prognosis in multiple cancers (47).	Preclinical; no approved agent specifically targeting NRF2 in BCSCs. NRF2 remains a promising but challenging target due to its dual role in tumor suppression versus promotion, limited specificity of current inhibitors, potential normal tissue toxicity, and compensatory activation of alternative redox pathways.
	HIF-1α signaling	HIF-1α, PHD, VEGFR, GLUT1, NQO1, TRX1, PHGDH	Metabolic shift to glycolysis; antioxidant enzyme upregulation; hypoxia adaptation; positive feedback with NRF2	Hypoxia activates HIF-1α, which upregulates GSTs, SOD, catalase (52, 53); HIF-1α and NRF2 form positive feedback loop (54); HIF-1α promotes glycolytic shift and serine synthesis via PHGDH (55); HIF-1α upregulates microRNA-1275 and activates Wnt/β-catenin and Notch pathways to maintain BCSCs self-renewal	HIF-1α expression correlates with poor prognosis and radioresistance; HIF inhibitors (digoxin, acriflavine, EZN-2968) in Phase I/II trials for advanced cancers; no BCSCs-specific clinical application.	Preclinical for dual targeting; HIF-1α/NRF2 axis is a promising strategy (54). Hypoxia-activated prodrugs in clinical development with limited success; no approved agent and no BCSCs-specific application.
	EMT/stemness acquisition	Snail, ZEB1, vimentin, N-cadherin, E-cadherin, TGF-β, NF-κB, Notch, Wnt, Hedgehog	Acquisition of migratory/invasive phenotype; non-stem cell → BCSCs conversion; enhanced radioresistance; self-renewal	RT induces EMT via ROS activating Snail, HIF-1, ZEB1, STAT3 within TGF-β, Wnt, Notch, PI3K/Akt, MAPK pathways (63, 65–69); ACTN4 induces EMT via Akt pathway (70); TGFβ-induced EMT generates BCSC-like cells with radioresistance (73); Notch and NF-κB upregulate Snail, conferring stemness to non-stem cells (35, 59).	Alpha-lipoic acid inhibits TGF-β signaling and NF-κB nuclear translocation, enhancing radiosensitivity (72); TGF-β inhibitors (galunisertib, fresolimumab, vactosertib) in clinical trials (76, 77).	Clinical investigation; TGF-β targeting in Phase I/II across solid tumors; vactosertib suppresses RT-induced fibrosis; EMT-BCSCs interplay closely linked to radioresistance (74).
TME-Mediated Resistance	CAFs-ECM remodeling	YAP/TAZ, TGF-β, HGF-c-Met, α-SMA, FAP, collagen I, fibronectin, hyaluronic acid	Dense ECM; physical barrier to CTLs; anti-apoptotic signaling; hypoxia maintenance; increased interstitial pressure	CAFs regulate collagen deposition leading to ECM linearization (78); dense ECM impedes oxygen diffusion, creating chronic hypoxia (92); ECM hardness activates Hippo/YAP/TAZ, upregulating BRCA1, RAD51 (94); surviving CAFs undergo senescence with SASP expressing HGF, CXCL12, EGF (95–97).	FAP-targeted PET/CT imaging in BC patients; FAP-TRT emerging for CAFs modulation (95, 103); TGF-β inhibitors reduce CAFs activation; PDGFR inhibitors (imatinib) under investigation.	Preclinical/early clinical; FAP^+^ CAFs as immunotherapy biomarker; CAFs-targeting strategies include FAP vaccines, CAR-T, and small molecule inhibitors (104).
	CAFs metabolic coupling	MCT1/MCT4, lactate dehydrogenase, RWE, OXPHOS	Lactate fueling of BCSCs OXPHOS; ATP generation for DDR; metabolic symbiosis; ROS scavenging	CAFs exhibit RWE (oxidative glycolysis), producing lactate transported to cancer cells via MCTs (92); cancer cells use lactate for OXPHOS, generating ATP for DDR (97); CAFs secrete GSH and ascorbic acid to clear ROS (99).	AZD3965 (MCT1 inhibitor) in Phase I clinical trials (NCT01791595); MCT1 inhibition enhances radiosensitivity by reducing lactate transport.	Phase I completed; MCT4 inhibitors (AZD0095) in clinical development; metabolic coupling represents emerging therapeutic target.
	Immunosuppression	PD-1/PD-L1, CTLA-4, CXCL12, TGF-β, IL-6, IL-10, CCL2, CXCL1	T-cell exhaustion; Treg/M2-TAM recruitment; MDSC expansion; immune evasion; CSCs maintenance	CAFs secrete TGF-β, CXCL12, IL-6 recruiting Tregs, M2-TAMs, MDSCs (95, 215); CAFs deposit ECM hindering cytotoxic T lymphocytes (CTLs) infiltration (“cold tumor”) (216); CAFs express FasL and PD-L1 inducing CTLs apoptosis/exhaustion (104); BCSCs upregulate PD-L1, activating PI3K/Akt while blocking TCR signaling (83); TAMs interact with CSCs activating Src/NF-κB (87); MDSCs regulate BCSCs via IL-6/STAT3 and NO/Notch (86).	Pembrolizumab approved for TNBC (KEYNOTE-522); atezolizumab + nab-paclitaxel (IMpassion130) (89); RT + pembrolizumab in TNBC (P-RAD, NCT04443348) (90); PD-L1 predictive value in BC heterogeneous (89).	FDA-approved for TNBC; RT + ICIs combination under active investigation; immunosuppressive microenvironment limits efficacy in “cold tumors” (91).
	Hypoxia-BCSCs crosstalk	HIF-1α, NRF2, xCT (SLC7A11), PHD	Sustained low ROS; metabolic adaptation; ferroptosis evasion	CAFs-deposited ECM creates hypoxia (92); HIF-1α/NRF2 feedback loop and downstream mechanisms detailed in Section 2.2.2 (41, 48, 51–56).	Hypoxia-activated prodrugs in clinical development; no approved BCSCs-specific agent.	Preclinical/early clinical; dual-axis targeting promising but clinically unvalidated (54).
Combination Strategies	RT + ICIs	PD-1/PD-L1, CTLA-4, CXCL16-CXCR6, CD8^+^ T cells, IFN-γ	Enhanced CD8^+^ T-cell infiltration; IFN-γ-mediated BCSC killing; abscopal effect; conversion of “cold” to “hot” tumors	RT induces ICD releasing tumor antigens (166); RT upregulates CXCL16, recruiting CXCR6^+^ T cells (159); RT + ICIs in TNBC model upregulates CD8^+^ T-cell infiltration, decreases MDSCs (157); RT + ICIs significantly reduces ALDH^+^ BCSCs and delays local recurrence (158); DNA damage + IFN-γ induces lethal oxidative stress in BCSCs (161); RT combined with CXCL13 induces tertiary lymphoid structures.	P-RAD trial (NCT04443348) investigating concurrent vs sequential RT + pembrolizumab (90); real-world safety data: grade 1 dermatitis higher with combo (83.3% vs 43.9%) (163); optimal dose/fractionation: 8–10 Gy×3 from preclinical (164); alternative regimens (7.5 Gy×1, 2 Gy×5) in trials (165).	Phase II/III ongoing; dermatitis and cardiopulmonary toxicity concerns (163, 168); abscopal effect observed in 4T1 model (160); PD-L1 predictive value heterogeneous (89).
	RT + BCSCs-targeted (stemness pathways)	Wnt/β-catenin (ATRA, vantictumab), Notch (GSI: MK-0752, RO4929097), Hedgehog (vismodegib)	BCSCs differentiation; delayed recurrence; reduced self-renewal; enhanced radiosensitivity	ATRA inhibits Wnt/β-catenin, reducing ALDH^+^ BCSCs by ~70% (173); Notch inhibition with GSIs reduces BCSCs ratio (127, 128); Wnt inhibition decreases BCSCs proliferation (130, 131); Hh inhibition increases BCSCs responsiveness to paclitaxel (136).	Vantictumab + paclitaxel in HER2- metastatic BC: Phase Ib, ORR 31.3%, fractures limit development (NCT01973309); MK-0752 + docetaxel: decreased CD44^+^/CD24^-^ cells (128); RO4929097 + exemestane in ER+ metastatic BC: Phase Ib (NCT01149356); Vismodegib: well tolerated but did not meet primary endpoint.	Phase I/II; limited efficacy due to CSC plasticity and normal stem cell toxicity (121, 176, 190); ATRA + RT in TNBC model shows significant recurrence delay (173).
	RT + metabolic targeting	Methionine restriction, PARP (olaparib, niraparib), EMSY, S-adenosylmethionine	Selective BCSC depletion + proliferating cell targeting; synthetic lethality; enhanced radiosensitivity	Methionine-restricted synchronized RT selectively clears BCSCs; PARP inhibitors target proliferating cells; combination achieves 80% tumor regression in PDX model; Methionine is metabolic dependency of tumor-initiating cells; EMSY-mediated methionine metabolism as therapeutic target (174, 217).	Olaparib + RT in TNBC (RadioPARP, NCT03109080): Phase I, well tolerated, 3-year EFS 65%; Olaparib + durvalumab in germline BRCA-mutated metastatic BC (MEDIOLA): Phase I/II (39); Niraparib + pembrolizumab (TOPACIO): Phase II, ORR 21% (47% in BRCA-mut) (43); Olaparib ± durvalumab (DORA): Phase II in pretreated TNBC (190).	Phase I/II; feasibility and tolerability under evaluation; PARP inhibitors combined with ICIs show promise.
	Nanodelivery & PROTAC	PROTAC (SHP2, BRD4, PD-L1), P-selectin, lipid nanoparticles, exosomes	Dual pathway inhibition; enhanced focal drug accumulation; targeted protein degradation; spatiotemporal coordination	P-selectin-mediated nanodelivery co-loads PARP inhibitor + PD-L1 inhibitor in BRCA1-deficient tumors (157); Radiation-induced P-selectin upregulation promotes targeted delivery to metastases (158); PROTAC NPRO targets SHP2 while inhibiting CD47/SIRPα and PD-1/PD-L1 (180); BRD4-targeted PROTAC modulates PD-L1 and c-Myc (181); Carbon-dot-based PD-L1-targeted PROTAC under investigation (182); Exosome-coated silica nanoparticles co-deliver 3,3’-diindolylmethane and doxorubicin (204).	mRNA lipid nanoparticles (NCT03739931): Phase I in advanced solid tumors/lymphoma (including BC) evaluating OX40L, IL-23, IL-36γ; ER-targeting PROTACs in HR+ BC (Phase I, ASCO 2026); Hyaluronic acid-modified nanoparticles co-deliver CRISPR/Cas9 + chemotherapy (40, 41).	Phase I; *in vivo* delivery, pharmacokinetics, and long-term safety remain barriers (177); PROTAC no FDA-approved agent yet.
	Gene editing (CRISPR-Cas9)	CXCR4/CXCR7, FBXO44, UBR5, MELK, epigenetic modifiers	Reversal of stemness; EMT inhibition; identification of therapeutic vulnerabilities; targeted gene knockout	CRISPR-Cas9 knockdown of CXCR4/CXCR7 impairs TNBC migration and growth (186); UBR5 knockout inhibits TNBC growth and metastasis (187); Hyaluronic acid-modified core-shell nanoparticles co-deliver CRISPR/Cas9 + chemotherapy, downregulate FBXO44 to revert BCSCs to normal tumor cells (40); System useful for non-coding RNA loss-of-function studies.	CRISPR-Cas9 screening for prognostic signatures in TNBC; multiple clinical trials for biomarker detection; *in vivo* delivery and off-target safety remain major barriers (184, 185); potential for ex vivo editing and CAR-T enhancement.	Preclinical/early clinical; *in vivo* delivery, off-target effects, and immunogenicity barriers remain (184, 185); promising for identifying therapeutic targets.

#### 6.1.1 Why BCSCs-targeted therapies have failed to deliver

Although preclinical studies have confirmed that BCSCs play a crucial role in treating drug resistance, therapies targeting BCSCs have failed to be continuously translated into clinical applications. Multiple phase II/III clinical trials targeting BCSCs-related pathways (such as the Notch inhibitor PF-03084014 and the Hedgehog inhibitor Vismodegib) were terminated due to failure to meet primary endpoints, side effects, or lack of efficacy (121). This difficulty in translation stems from a series of interrelated obstacles.

First, targeted therapy is limited by the plasticity of BCSCs. As a central component of tumor heterogeneity, BCSCs exhibit a high degree of intratumoral and intertumoral plasticity, and differences in their surface marker expression profiles and activation status of key signaling pathways significantly increase the difficulty in identifying universal therapeutic targets. BCSCs are not a fixed cell population but a functional state. Non-stem cancer cells can differentiate and regain stemness through epigenetic reprogramming or microenvironmental induction under therapeutic stress (188). This dynamic plasticity means that even if the existing BCSCs are successfully eliminated by the therapy, the residual non-stem cancer cells can redifferentiate and regain stem cell-like characteristics through epigenetic reprogramming or microenvironmental signals. This plasticity makes patient screening strategies based on static biomarkers inapplicable - a patient may be identified as “low BCSCs” at baseline but may become “high BCSCs” after the start of treatment (116). The key to therapeutic breakthroughs lies in developing comprehensive strategies that simultaneously target both BCSCs and the majority of non-stem cancer cells that make up the bulk of the tumor, to block the stemness replenishment chain and prevent acquired resistance, thus laying the groundwork for durable clinical efficacy (127).

Second, preclinical models are difficult to truly simulate the biological characteristics of BCSCs in the human body. Most BCSC-targeted drugs show significant efficacy in immunodeficient mouse xenograft models. However, this model can’t simulate the complex human TME, especially the immune components that play a key regulatory role in BCSC behavior (189). Additionally, mouse studies typically observe tumor growth over several weeks, which cannot simulate the long latency period of tumor recurrence driven by BCSCs, which often occurs several years after treatment (175).

Third, toxicity to normal stem cells. BCSCs have the same key signaling pathways as normal tissue stem cells (such as breast stem cells, hematopoietic stem cells, intestinal crypt stem cells): Wnt, Notch, Hedgehog, etc. (12). The dose-limiting toxicities observed in clinical studies, such as the gastrointestinal toxicity of Notch inhibitors (176) and the myelosuppression of Hedgehog inhibitors (190), precisely stem from the indispensability of their target pathways in the normal functions of stem cells, revealing the inseparability of efficacy and toxicity in a biological sense. The “treatment window” problem of avoiding damage to normal stem cells while killing BCSCs is far beyond the estimation of preclinical studies.

Fourth, the lack of validated efficacy biomarkers hinders the rational design of clinical trials. Without reliable biomarkers to confirm that the drug targets and acts on BCSCs in patients, failure of the trial can’t distinguish whether it is “the drug is ineffective against the target” or “the target itself is unrelated to clinical response” (191). Currently, BCSCs’ detection methods exhibit significant inter-laboratory variation and lack standardized thresholds, rendering them unsuitable for implementation in multicenter trials.

Fifth, most trials only target BCSCs, rather than combined treatment. BCSCs account for only 0.1%–1% of tumor cells, and eliminating BCSCs without simultaneously controlling the growth of the main tumor may not translate into clinical benefits (113). The failure to combine BCSC-targeted drugs with effective cell ablation therapy may be a key reason for the negative results of the clinical trial.

#### 6.1.2 Limitations of RT-immunotherapy combinations

Although immunotherapy has become an important pillar of oncology, its clinical benefits vary significantly among patients. Fluctuations in efficacy are closely related to individual genetic background, baseline immune function, and the immune profile of the TME; at the same time, immune-related adverse events (irAEs), including autoimmune phenomena and cytokine-release syndromes, are heterogeneous in terms of frequency and severity, which can weaken adherence to treatment and deteriorate prognosis in critically ill patients (163, 192).

The combination of RT and ICIs theoretically enhances anti-tumor immunity by inducing ICD and triggering distant effects, in clinical practice for BC, but this strategy still faces multiple challenges, and the benefit-to-risk ratio has not been fully defined. Firstly, the safety of the combined treatment still needs to be carefully evaluated. Although preclinical studies suggest good safety, a recent retrospective clinical study showed that in 89 patients with TNBC who received both pembrolizumab and adjuvant RT, the incidence of grade 1 radiation-induced dermatitis in the combined treatment group was significantly higher than that in the control group (83.3% vs 43.9%), and only 2 cases of grade 1 pulmonary toxicity reactions were observed in the combined group. Due to the short median follow-up time of only 16 months, the long-term risk of radiation-induced cardiopulmonary toxicity, especially the potential synergistic cardiac toxicity from the combination of RT and pembrolizumab (which itself has a potential risk of myocarditis), still needs to be clarified through longer-term follow-up (168). Secondly, although preclinical studies suggest that in the mouse model of TNBC, simultaneous administration of RT and anti-PD-1 treatment is superior to sequential or single therapy in activating systemic immunity and inhibiting tumors (193), this conclusion may not apply to humans and needs to be verified. The ongoing P-RAD trial (NCT04443348) is exploring differences in efficacy between concurrent and sequential combinations in BC. Furthermore, the optimal RT dose and fractionation schedule to effectively stimulate anti-tumor immunity in BC patients remain undefined. In previous preclinical studies, the optimal RT dose and fractionation schedule for immune activation were typically 8–10 Gy×3 fractions (164). Later, evidence indicated that this protocol’s efficacy in preclinical settings might not directly translate into clinical effects when combined with immunotherapy (194). Clinical trials also explored alternative lower-dose regimens (such as 7.5 Gy×1 fraction or 2 Gy×5 fractions) as immune activation strategies (165). Unlike melanoma or non-small cell lung cancer, the predictive value of PD-L1 in BC (especially when combined with RT) is unclear and heterogeneous (NCT02425891) (89); and microsatellite instability (MSI-H) is relatively rare in BC and has limited clinical application (195). The absence of biomarkers makes it impossible to precisely screen the benefit population, resulting in some patients bearing unnecessary toxicity risks without clinical benefits, seriously hindering the rational application and efficacy optimization of this combined strategy. Most BC, especially the hormone receptor-positive subtype, belong to immunologically “cold tumors”, characterized by scarce lymphocyte infiltration within the tumor and the enrichment of various immunosuppressive cells (91). This microenvironment may weaken the efficacy of RT in converting “cold tumors” into “hot tumors”, making the induced immune response more susceptible to suppression by the inhibitory microenvironment, thereby limiting synergy with ICIs (196).

Different subtypes exhibit markedly variable responses to RT-ICIs combinations. TNBC, with its high TMB, TILs, and PD-L1 expression, is the primary beneficiary population, yet even within this subtype, the predictive value of PD-L1 expression remains heterogeneous (NCT02425891) (89). In HER2+ subtypes, HER2-targeted therapy itself modulates the immune microenvironment, but MSI-H is relatively rare in BC, limiting this biomarker’s applicability. Luminal-type BC, as a typical “cold tumor” with sparse lymphocytic infiltration and abundant immunosuppressive cells (Tregs, M2-TAMs), may attenuate RT-induced ‘cold-to-hot’ conversion, thereby limiting ICI synergy. Therefore, subtype-specific patient selection and combination strategy optimization are critical for improving RT-ICIs’ efficacy.

Furthermore, the optimal combination regimen of RT and ICIs remains unclear. Some studies have used mathematical modeling to predict that simultaneous use of anti-PD-1/PD-L1 therapy with RT may yield the strongest anti-tumor immune response. However, this prediction urgently needs to be verified through prospective clinical trials. The ongoing P-RAD trial (NCT04443348) is designed to address this issue by comparing the efficacy of concurrent RT plus pembrolizumab versus sequential RT plus pembrolizumab in BC. Nevertheless, even this trial is limited to the neoadjuvant setting and may not be applicable to adjuvant or metastatic settings.

From the perspective of regulation and implementation, the development path of multimodal combined treatment involving RT, systemic drugs, and new drug delivery carriers remains unclear. Traditional oncological endpoints [such as objective response rate (ORR) and progression-free survival (PFS)] are difficult to capture biological effects such as the elimination of BCSCs, remodeling of the microenvironment, or immune memory, and these effects may be more clinically significant than mere tumor regression. There is an urgent need to establish a composite endpoint indicator that integrates pathological complete response, clearance of circulating tumor cells/circulating tumor DNA, and immune cell infiltration scores to accurately assess the true clinical value of BCSCs-TME-targeted therapy.

#### 6.1.3 Unresolved questions in the clinical translation of BCSC-TME targeting

The limitations articulated in Sections 6.1.1 and 6.1.2 converge on several fundamental questions that must be resolved to advance BCSCs-TME targeted radiosensitization from mechanistic promise to clinical reality.

At the level of target specificity, a critical unresolved issue is whether PARP inhibitors selectively eradicate radioresistant BCSCs or merely exploit homologous recombination deficiency across the entire tumor cell population—current trials such as RADIOPARP (NCT03109080) and TOPACIO (NCT02657889) were not designed with BCSC-specific endpoints, leaving this distinction unresolved. Closely related is the therapeutic window for sparing normal stem cells: NRF2, Notch, and Hedgehog pathways are essential for hematopoietic and intestinal crypt stem cells, yet the dose range achieving BCSC-selective inhibition without unacceptable myelosuppression or mucositis has not been defined in any clinical trial to date.

With respect to treatment sequencing and combination strategies, while preclinical models suggest that concurrent administration of RT and ICIs maximizes the “immune sensitization window,” overlapping toxicities—particularly radiation pneumonitis and myocarditis—may offset these benefits. The P-RAD trial (NCT04443348) is prospectively addressing this in the neoadjuvant setting, but adjuvant and metastatic scenarios remain unexplored. Similarly, although TGF-β inhibitors reverse EMT and sensitize BCSCs to RT in preclinical models, cardiac fibrosis and impaired wound healing have limited their clinical translation; whether alternative targets—such as ZMYND8 epigenetic modulation or isoform-specific EMT transcription factor inhibition—can achieve BCSCs-selective EMT suppression without disrupting normal tissue homeostasis remains unknown. Subtype-specific prioritization of CAFs-targeting strategies also remains undefined: CAFs metabolic coupling via MCT1/MCT4 dominates in TNBC, whereas TGF-β/ECM remodeling prevails in Luminal-type disease, yet it is unclear whether MCT1 inhibitors (AZD3965) or TGF-β inhibitors should be prioritized by subtype in RT combinations, or whether a unified CAFs-depletion strategy (e.g., FAP-targeted) is preferable.

In the domain of biomarkers and dynamic monitoring, no validated composite biomarker currently exists for predicting RT-ICIs response across BC subtypes. PD-L1, TMB, and ctDNA dynamics each show subtype-dependent predictive value, with PD-L1 demonstrating particularly poor utility in hormone receptor-positive disease—yet current trials do not stratify by subtype-specific biomarker profiles. Furthermore, the spatiotemporal dynamics of TAM polarization have been characterized in cervical cancer and glioblastoma, but whether analogous spatial redistribution occurs in BC recurrence remains unknown; direct extrapolation is currently unsupported and requires longitudinal spatial transcriptomic studies in BC-specific cohorts. Finally, current biomarkers such as ALDH1 and CD44/CD24 are static and fail to capture the phenotypic plasticity of BCSCs during fractionated RT. Real-time, non-invasive monitoring of BCSCs state transitions—potentially via ctDNA methylation signatures or exosomal proteomic trajectories—remains technically elusive and clinically unvalidated.

Strategic implication: Addressing these questions requires a paradigm shift in trial design: biomarker-driven enrollment with BCSC-specific co-primary endpoints (e.g., circulating tumor cell stemness score, post-treatment ALDH1^+^ cell clearance), adaptive dosing based on dynamic biomarker feedback, and regulatory frameworks accommodating multi-modal endpoint assessment beyond conventional ORR/PFS metrics.

### 6.2 Future directions and innovative approaches

#### 6.2.1 Single-cell sequencing to decipher the dynamic interactions between BCSCs and immunity

Single-cell sequencing technology enables high-resolution profiling of individual tumor cells, providing unprecedented precision for dissecting the heterogeneity of BCSCs and their networks of interactions with immune cells (197). The newly established high-throughput single-cell multi-omics platform UDA-seq can simultaneously capture BCSCs’ surface markers, transcriptional status, and information on neighboring immune cells in a single experiment, enabling system-level observation of BCSCs-immune cell interactions. Similar strategies have successfully targeted rare immunosuppressive subpopulations and mapped their intercellular communication in esophageal squamous cell carcinoma (ESCC) (198, 199), providing a readily transferable technical framework for BC research. By precisely identifying the key ligand-receptor pairs and downstream signaling axes that drive the dialogue between BCSCs and immune cells, these approaches will provide a solid molecular foundation for therapeutic strategies targeting the BCSCs’ microenvironment.

#### 6.2.2 Artificial Intelligence (AI) for predicting the risk of radioresistance and personalized treatment

AI constructs quantifiable radioresistance prediction models by integrating and analyzing multidimensional data such as genomic profiling, imaging, histology, and treatment history, thereby providing an important basis for the development of individualized treatment regimens by facilitating the combined application of radiation therapy and other treatment modalities to overcome drug resistance (200). Machine learning models that integrate imaging histology with clinical biomarkers, such as CT-based radiology scores and HER2 status, have achieved up to 85.9% accuracy in predicting lymph node metastasis in gastric cancer (201). A similar technique can be used to risk-stratify BC patients based on tumor cell abundance and TME features to optimize RT regimens (202).

#### 6.2.3 Clinical translation of multimodal therapeutic strategies

Single-cell sequencing and AI prediction models provide a foundation for identifying targets and screening populations. However, for multimodal treatment strategies targeting BCSCs and the TME, the transition from the laboratory to clinical practice faces a series of unique, more complex systemic obstacles.

##### 6.2.3.1 Preliminary exploration of early clinical trials

Currently, clinical translation mainly focuses on evaluating combined treatment regimens involving RT with targeted therapy or immunotherapy. Multiple I/II phase trials aim to assess their safety and initial efficacy. For example, the I/II phase trial of olaparib combined with RT for TNBC (NCT03109080) reported controlled toxicity, establishing preliminary safety data for this combination (39). In the field of immunotherapy, pembrolizumab combined with RT has shown potential to induce distant effects (203), and the ongoing P-RAD trial (NCT04443348) aims to evaluate its role in neoadjuvant therapy. Additionally, clinical trials such as TOPACIO/KEYNOTE-162 (NCT02657889) (which includes a TNBC cohort) (43) and DORA (NCT03167619) (40) have combined PARP inhibitors with ICIs. The preliminary results of these trials indicate that multimodal treatment for BC has moved from purely empirical approaches to clinical validation based on rationally designed targeting mechanisms. These trials provide crucial clinical references for subsequent combined treatment schemes that incorporate more diverse methods, such as RT and targeted metabolic drugs, including information on dosages, safety, and initial efficacy.

##### 6.2.3.2 Nanodelivery technology for achieving targeted synergy

To achieve more precise spatiotemporal coordination, nanocarrier-based co-delivery platforms have become a key technology. Preclinical studies have demonstrated that nanocarriers can simultaneously deliver metabolic inhibitors and ICIs, achieving dual targeting of BCSCs. For instance, hyaluronic acid-modified core-shell nanoparticles can simultaneously deliver the CRISPR/Cas9 system and chemotherapeutic agents. By downregulating the expression of FBXO44, they can revert BCSCs to normal tumor cells, thereby effectively inhibiting the growth of TNBC (41). Moreover, the exosome-coated mesoporous silica nanoparticles can jointly deliver 3,3’-diindolylmethane and doxorubicin, thereby significantly reversing the EMT transition driven by BCSCs (204). Currently, this technology has entered the clinical translation stage. In TNBC models, nanoparticle-based co-delivery platforms synchronized the release of methionine-restricted metabolism inhibitors with ICIs to achieve dual metabolic-immune blockade, thereby significantly inhibiting the self-renewal of BCSCs and prolonging tumor-free survival (174, 205). In addition, an I-phase trial (NCT03739931) is evaluating lipid nanoparticles loaded with mRNA encoding immune-regulatory factors (OX40L, IL-23, IL-36γ), marking the first clinical attempt to integrate nanotechnology, immunotherapy, and gene therapy. However, the clinical pharmacokinetics, tumor internal distribution, and long-term safety of such complex formulations still need to be fully verified in humans.

##### 6.2.3.3 The core challenge of clinical translation: the leap from mechanism to practice

The primary scientific challenge in translating preclinical-effective multimodal strategies into standard clinical therapies lies in optimizing the treatment plan in both time and space. The sequence, timing window, and dose intensity of different therapies (radiation, immunotherapy, targeted therapy) have a decisive impact on the final efficacy and toxicity profile. For instance, the simultaneous application of RT and ICIs can theoretically maximize the induction of ICD and activate systemic anti-tumor immunity, but it also significantly increases the risk of overlapping toxicities such as radiation pneumonitis and myocarditis; while sequential administration may miss the transient “immune sensitization window” created by RT, thereby weakening the synergistic effect (206). Mathematical modeling studies suggest that the simultaneous administration of anti-PD-1/PD-L1 therapy with radiation may yield the optimal effect, but this prediction urgently requires validation through prospective clinical trials (90). Currently, there is a lack of high-level evidence regarding the optimal timing sequence, radiation fractionation scheme, and target area range for the combination of the two in the field of BC, and the existing data mostly come from small-sample exploratory studies of I/II phases (207). In the context of multi-drug combination therapy, the traditional dose-escalation model is not applicable, and adaptive trial design and pharmacokinetic/pharmacodynamic models need to be employed to find a new balance between efficacy and toxicity.

Secondly, there is a lack of reliable dynamic biomarkers to guide the dynamic adjustment of treatment. Although single-cell sequencing can high-resolutionly analyze the heterogeneity of BCSCs before treatment, AI models can predict the initial radiation resistance risk, but there is still a lack of technical means in clinical practice that can non-invasively, in real-time, and dynamically monitor the clonal evolution of BCSCs, the remodeling of the immune microenvironment, and the treatment pressure during the treatment process. Liquid biopsy techniques such as circulating tumor DNA and the exosomal proteome show potential, but their sensitivity, specificity, and standardized procedures for real-time guidance of dynamic adjustment of multimodal therapy still need to be established through prospective research (208, 209).

At the level of the regulatory and evaluation system, the regulatory path for multimodal combination therapy—integrating RT, systemic agents, and novel delivery systems—remains ill-defined (210, 211). Traditional endpoint indicators based on tumor shrinkage on imaging (such as ORR, PFS) are difficult to comprehensively capture key biological effects, such as BCSCs clearance, microenvironmental remodeling, and treatment pressure (212). Therefore, it is urgent to establish a new composite endpoint that integrates pathological complete response, clearance of circulating tumor cells/ctDNA, and immune cell infiltration scores to more accurately evaluate the true clinical value of such therapies.

Finally, at the practical application level, integrating single-cell sequencing, AI prediction, and clinical decision-making to achieve a closed loop of “mechanism analysis - risk prediction - plan implementation” poses significant challenges in terms of technology, data, and cost for most current medical centers (213, 214).

## 7 Conclusions

In conclusion, this review systematically delineates the BCSCs-TME axis as the biological basis of acquired radioresistance in BC, integrating preclinical mechanistic insights with clinical translational evidence to address three critical knowledge gaps. First, we highlight the subtype-specific heterogeneity of BCSCs-TME interactions: TNBC is characterized by NF-κB/Wnt-driven DNA repair enhancement and MDSC/TAM-mediated immunosuppression; HER2+ subtypes exhibit persistent HER2-PI3K-Akt-mTOR activation and CAFs-HGF/c-Met signaling; while Luminal-type disease relies more on endocrine signaling and cyclin dysregulation—distinctions that necessitate precision combination strategies. Second, we critically appraise the spatiotemporal dynamics of RT-induced microenvironmental remodeling, emphasizing that TAM reprogramming follows a biphasic temporal pattern and that BCSCs occupy distinct spatial niches, demanding temporally optimized therapeutic interventions. Third, we identify the profound evidence-to-practice gap: preclinically validated targets such as NRF2 and CAFs-directed therapies lack clinical biomarkers and approved agents, while clinically established therapies (ICIs, PARP inhibitors) have not been proven to specifically eradicate radioresistant BCSCs. By explicitly distinguishing evidence levels, identifying contradictory findings, and outlining structural limitations of clinical trial design for drug-RT combinations, this review provides a framework for rational trial design. Future progress will depend on integrating single-cell multi-omics, AI-driven predictive models, and dynamic biomarkers (ctDNA, exosomal proteomics) to achieve a closed loop of ‘mechanism analysis—risk prediction—treatment implementation,’ ultimately translating the BCSCs-TME paradigm into precision RT for BC patients.

## Data Availability

The raw data supporting the conclusions of this article will be made available by the authors, without undue reservation.
